# GAMA: A Robust and Automated Fragment-Based Quantum Chemistry Method for Biomolecular Systems

**DOI:** 10.1021/acs.jpclett.5c03778

**Published:** 2026-03-18

**Authors:** Sujan Kundu, Arjun Saha

**Affiliations:** Department of Chemistry and Biochemistry, University of Wisconsin—Milwaukee, Milwaukee, Wisconsin 53211, United States

## Abstract

Accurate quantum chemical treatment of covalently bonded biomolecules using fragment-based approaches remains a major challenge as fragmenting across covalent bonds disrupts essential electron correlation and long-range polarization. Importantly, a few of the previously developed fragment-based methods can accurately and efficiently treat both noncovalently and covalently bonded molecular systems, highlighting a significant gap in the field. A key novelty of the grid-adapted many-body analysis (GAMA) framework is that it overcomes this limitation. Building on our earlier work establishing GAMA for noncovalent systems, we extend this framework to covalently bonded biomolecules and develop GAMA2, a fully automated protocol that integrates a simple grid-based fragmentation scheme, many-body expansion with overlapping fragments truncated at two-body order, and a multilayer low-level correction. Across diverse peptides, ranging from flexible bioactive motifs to structured 18-mer helixes, GAMA2 reproduces supersystem MP2/6-311G­(d,p) energies with unsigned absolute errors of ∼0.01–4 kcal/mol for flexible small- and medium-size peptide systems using HF as a low level of theory and ∼2–5 kcal/mol for complicated helical-type peptide structures when using M06-2X/6-311G­(d,p) as the low-level method, showing substantial improvement over HF using accurate DFT-based methods. In addition to this highly accurate results, GAMA2 also demonstrate a significant computational speedup with HF as a super system low-level method relative to the reference full MP2 calculation, establishing GAMA2 as a scalable, efficient, and systematically improvable route for correlated quantum chemical calculations on biomolecular systems.

## Graphical abstract

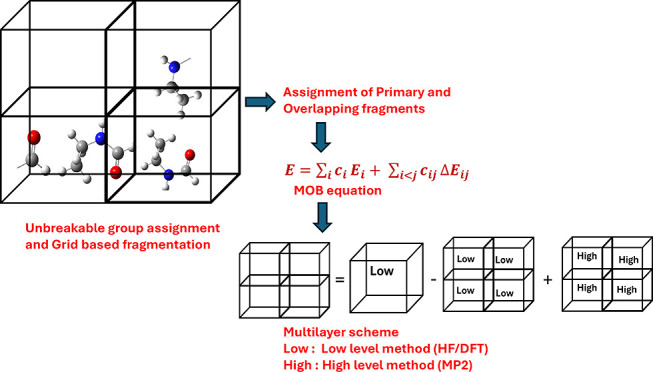

Fragment-based quantum chemistry (FBQC) methods^1–6^ continue to provide a powerful approach for extending correlated quantum mechanics to systems far beyond the reach of conventional MP2 or coupled-cluster calculations. These methods aim to recover the essential physics of large molecules by decomposing them into manageable subsystems, computing their energies independently and reconstructing the total energy using many-body expansions. Despite impressive progress, reliably treating systems with covalent connectivity remains a long-standing challenge as fragmenting across chemical covalent bonds disrupts orbital delocalization, local polarization, and correlation effects that must be accurately recovered. A general, automated, and physically motivated solution to this problem would greatly expand the applicability of fragment-based quantum chemistry to biomolecular systems.

In our previous work, we introduced the grid-adapted many-body analysis (GAMA) framework,^7^ along with its charge-embedded extension, EE-GAMA,^8^ to enable accurate and efficient fragment-based quantum calculations on molecular systems, particularly those dominated by noncovalent interactions. The original GAMA^7^ framework was systematically validated for noncovalent water clusters. In this approach, the entire system is first enclosed within a three-dimensional spatial box, which is subsequently partitioned into smaller grid cells. Each water molecule is treated as an indivisible unit to preserve its internal structure during fragmentation. Based on this grid partitioning, primary fragments are constructed from molecules residing within a given grid, while overlapping fragments are generated by including molecules at the interfaces of neighboring grids. When the primary and overlapping fragments are treated as monomeric units, their energies are evaluated within the many overlapping body expansion^9^ (MOBE) framework, truncated at the two-body term. To capture residual long-range many-body effects, a two-layer correction scheme, analogous to the MIM-type^10^ or ONIOM-type^11^ approach, is incorporated, enabling an accurate reconstruction of the total system energy from the fragment contributions. The EE-GAMA^8^ framework extends this methodology by introducing electrostatic embedding, wherein each fragment is computed in the presence of background point charges that represent the electrostatic influence of the surrounding molecular environment. This inclusion allows the fragment calculations to capture polarization and other environment-dependent effects that are otherwise neglected in non-embedded GAMA. EE-GAMA has been successfully applied to both neutral and protonated water clusters (hydronium cluster systems), demonstrating a significant enhancement in accuracy compared to the original, non-embedded GAMA scheme. With the systematic combination of fragment-based expansion with electrostatic embedding, EE-GAMA provides a robust and scalable approach for high-precision quantum chemical calculations of large noncovalently bonded molecular assemblies.

In this work, we generalize this approach to covalently bonded peptide systems. This extension preserves the simplicity and physical transparency of the original method while enabling grid-based fragmentation at correlated levels of theory. The major goal of this work is to develop a fully automated and systematically improvable FBQC framework applicable to covalently bonded biomolecular systems, where fragmentation across covalent bonds poses a fundamental challenge. Building on our earlier GAMA framework developed for noncovalently bonded water clusters, we extend the GAMA protocol to covalently bonded peptide systems. The approach combines a grid-based fragmentation scheme, a many-body expansion truncated at the two-body level, and a low-level correction. The major impact of this work is the demonstration that GAMA2 (GAMA with two different layers) provides a controllable and efficient FBQC approach whose performance can be systematically tuned through the choice of low-level theory, grid box size, and interaction distance cutoff, enabling accurate MP2-level energies at substantially reduced computational cost and significantly advancing fragment-based treatments of large biomolecular systems. The practical implementation of this GAMA framework for covalently bonded peptide systems is described as follows. To construct peptide fragments, the full structure is enclosed in a simulation box defined by its Cartesian extent and partitioned into smaller cubic grid cells. In parallel, the peptide backbone is segmented by cleaving the relatively less polar C–C single bond (across the peptide backbone) between C-α of residue *i* and carbonyl carbon of residue *i* + 1, with broken valencies saturated using standard hydrogen link atoms.^12–14^ This avoids cutting through the polar peptide bond and N-C-α bond, which eliminates the need for the more complex capping strategies used in MFCC-type^15,16^ schemes. Here, we also avoid cutting the C–C bond at the side chain of the peptide. Herbert and co-workers^14^ highlighted the advantages of cutting the C–C single bond between α carbon and carbonyl carbon along the peptide backbone while retaining the polar peptide and N–C-α bonds. Each “group of atoms” (unbreakable segment) is mapped onto the grid; groups occupying a given grid cell define primary fragments. Because a group may span adjacent cells, overlapping fragments arise naturally, an essential feature of GAMA. These primary and overlapping fragments constitute the monomers for the MOBE^9^ approach, truncated here at the two-body level to balance accuracy and efficiency. Long-range electrostatics are recovered via a multilayer ONIOM-like^11,17^ correction: fragment interactions are computed at the MP2/6-311G­(d,p) level within MOBE, while a full-system HF/6-311G­(d,p) calculation supplies complementary long-range contributions. This multilayer strategy, established in our earlier work,^7,8^ provides a systematically improvable treatment of both short- and long-range correlation effects. We refer to the present two-level formulation as GAMA2. Full methodological and mathematical details are provided in our earlier publications.^7,8^

GAMA2 adopts an overlapping, grid-defined fragmentation strategy combined with a multilayer framework to address challenges associated with covalently connected biomolecular systems. In contrast to cap-based approaches, such as molecular fractionation with conjugate caps^15,16,18^ (MFCC), the method avoids the introduction of artificial caps and the associated boundary effects. Relative to standard non-overlapping many-body expansion (MBE)-based schemes, the use of overlapping fragments within GAMA2 enables recovery of important delocalization, polarization, and correlation effects without requiring high-order expansions or very large embedding domains. While fragment molecular orbital (FMO) methods^19,20^ efficiently account for polarization, GAMA2’s grid-based construction generates overlapping fragments, enabling a more systematic treatment of electronic delocalization. Overall, GAMA2 is intended as an alternative fragmentation framework that emphasizes automation, systematic improvability, and compatibility with correlated wave function methods rather than as a direct comparison or direct replacement for other existing FBQC approaches. A chemically diverse set of peptide systems was selected to rigorously assess the performance of the GAMA protocol on covalently connected biomolecular structures. The benchmark includes short bioactive peptides, such as Segglu, Segtrp, and Tuftsin, which contain varied side-chain functionalities and localized polar environments. To probe systems with increased conformational flexibility and extended hydrogen-bond networks, we incorporated a medium-length glycine oligomer (Gly_12_) and the structured peptide 1YJP, a compact model system with well-defined secondary motifs. For further assessing the method’s performance across distinct regimes of backbone folding and intramolecular cooperativity, we examined three conformational isomers of alanine 18-mers corresponding to β-strand,α-helix, and 3_10_-helix topologies. All three (Ala)_18_ peptides are capped at the N terminus with an acetyl group and at the C terminus with an amide group [acetyl-(Ala)_18_-NH_2_]; for simplicity, they are hereafter referred to simply as (Ala)_18_. These larger systems differ markedly in packing density, hydrogen-bond periodicity, and extent of electronic delocalization along the peptide backbone. Collectively, this benchmark set spans a broad spectrum of structural compactness, dipolar organization, and correlation patterns, providing a stringent and representative testing ground for evaluating fragment-based QM treatments of covalently bonded peptides. Structures of all the peptide systems considered in this study are provided in Figure S1. Cartesian coordinates of all peptide systems considered in this study are provided in the Supporting Information, which were taken from ref 4 (please see the Supporting Information of ref 4).

GAMA2 performance with a 2 Å box size and a 5 Å cutoff radius is shown in Figure 1 (Table S1). For bioactive peptides and moderately extended structures, deviations from full MP2/6-311G­(d,p) results remain low (0.01–4 kcal/mol), indicating reliable treatment of flexible and moderately compact systems. Both Gly_12_ (error is 3.87 kcal/mol) and the β-strand-Ala_18_ conformer (error is 4.0 kcal/mol) fall in this regime, demonstrating good performance for elongated backbones with largely localized interactions. As expected, compact and hydrogen-bond-rich helical structures exhibit larger errors: α-helix-Ala_18_ shows a deviation of 13.53 kcal/mol, and 3_10_-helix-Ala_18_ yields a deviation of 7.66 kcal/mol, reflecting the difficulty of capturing long-range cooperative polarization within the MP2/HF-based multilayer correction.

**Figure 1:**
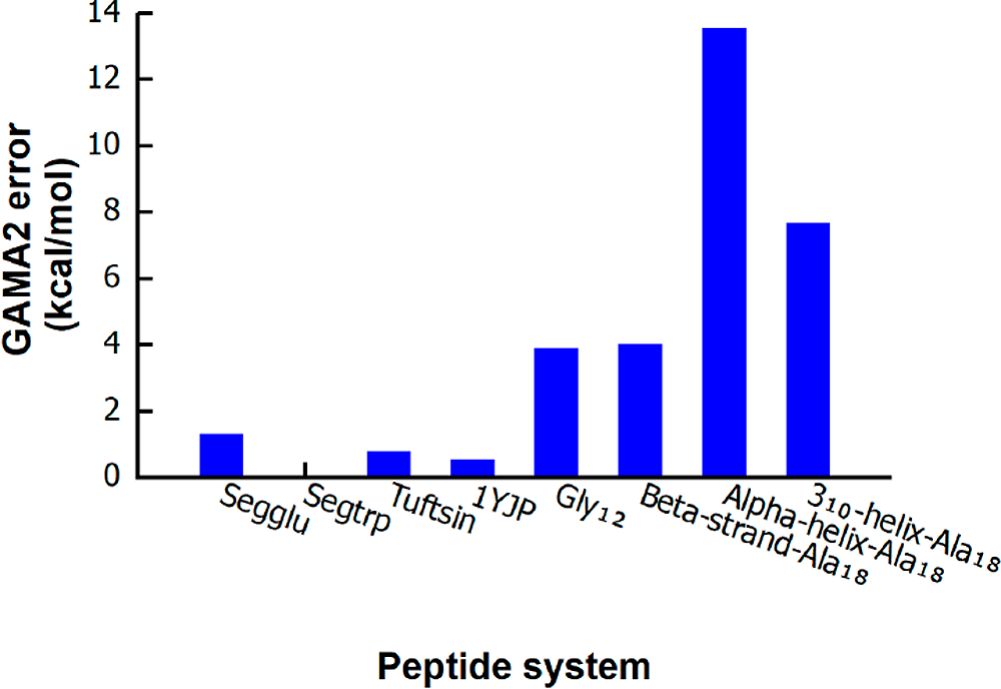
Absolute energy errors of GAMA2 for a diverse set of peptide systems relative to reference supersystem MP2 energies. All calculations were performed using a 2 Å box size and a 5 Å cutoff radius, with MP2/6-311G­(d,p) employed as a high-level theory and HF/6-311G­(d,p) employed as a low-level theory. In Segtrp, the GAMA2 error is ∼0.01 kcal/mol; because this value is extremely small, the corresponding blue bar is nearly invisible in the plot.

To probe this limitation, we examined the effect of improving the low-level method (Figure 2 and Table S2). Replacing HF/6-311G­(d,p) with B3LYP/6-311G­(d,p) or M06-2X/6-311G­(d,p) significantly reduces GAMA2 errors, especially for α-helix-Ala_18_ and 3_10_-helix-Ala_18_, medium-sized peptides (1YJP and Gly_12_), and β-strand-Ala_18_. These trends highlight the central role of accurate low-level corrections in achieving robust GAMA2 energies.

**Figure 2:**
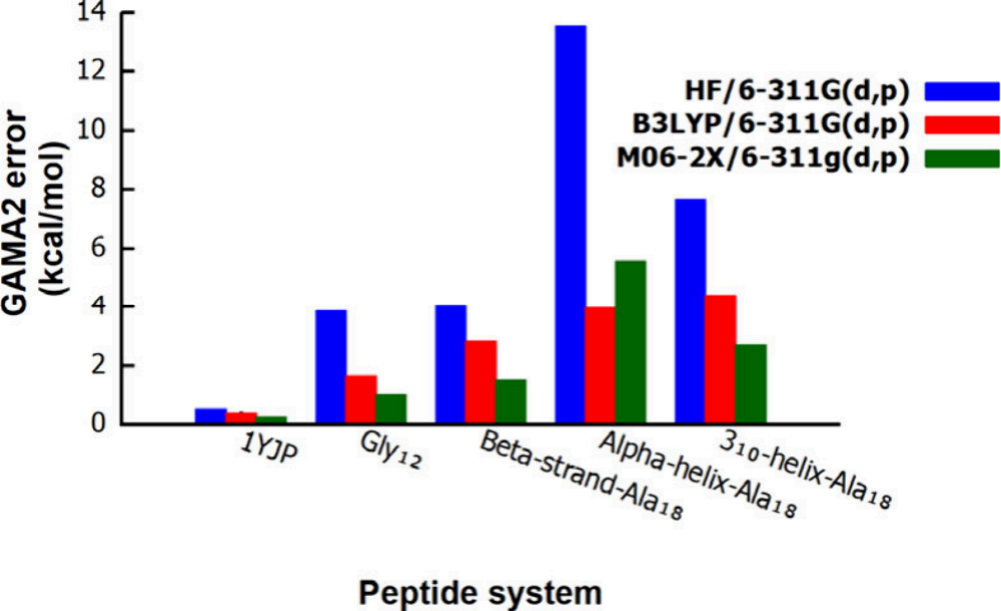
Influence of three low-level methods, HF/6-311G­(d,p), B3LYP/6-311G­(d,p), and M06-2X/6-311G­(d,p), on the absolute GAMA2 energy error across five peptide systems. All calculations employ a 2 Å grid-box size and a 5 Å cutoff radius with MP2/6-311G­(d,p) used as the high level.

Existing FBQC methods^1–3,6,16^ have laid the strong foundation of this field in the last 3 decades, providing an opportunity to make these methods more powerful. Toward that direction, we are focusing on existing challenges. Published benchmarks in the literature provide useful context regarding the challenges faced by FBQC methods for covalently bonded peptide systems. For example, Vornweg and co-workers^16^ applied conventional MFCC and MFCC-MBE(2) schemes to three Ala_10_ peptide isomers (α-helix, β-strand, and 3_10_-helix) and reported deviations from supersystem MP2 energies: MFCC errors of ∼60–80 kcal/mol (∼6–8 kcal/mol per residue) and MFCC-MBE(2) errors of ∼6–8 kcal/mol (∼0.6–0.8 kcal/mol per peptide) for the α-helix and 3_10_-helix isomers. Similar trends have been observed for FMO methods,^21^ which improve upon MFCC for polar or charged peptides but still show deviations of ∼10–16 kcal/mol (∼0.5–2 kcal/mol per residue) for FMO2 and ∼5–8 kcal/mol (∼0.2–0.8 kcal/mol per residue) for FMO3 in α-helical polyalanine chains of 10–20 residues. Systematic molecular fragmentation (SMF) and MBE-with-capping approaches also show deviations of ∼8–20 kcal/mol (∼0.8–2 kcal/mol per residue) for compact or α-helical structures, reflecting incomplete recovery of higher order polarization, charge-transfer, and cooperative hydrogen-bonding effects. These studies additionally highlight the considerable computational cost of several FBQC approaches,^1,4,6^ particularly higher-order MBE, FMO3, and large-cutoff SMF approaches, which can require hundreds to thousands of CPU hours for 15–20 residue peptides. Importantly, because these published benchmarks employ varying fragmentation protocols and reference levels of theory, they do not allow for a direct side-by-side comparison to GAMA. Consequently, we do not claim that GAMA offers superior accuracy relative to those established benchmarks. Instead, these literature results serve to contextualize the broader challenges within the field and provide a frame of reference for our method’s performance. Within this context, GAMA2 achieves total energy deviations of 0.01–4 kcal/mol for flexible peptides (HF low level) and 2–5 kcal/mol for highly cooperative α-helical-Ala_18_ and 3_10_-helical-Ala_18_ systems (DFT low level). Beyond accuracy, GAMA2 significantly optimizes efficiency: a full MP2 calculation for the α-helix-Ala_18_ peptide system requires ∼1178 CPU hours, whereas GAMA2 completes the task in just ∼77 CPU hours (Figure S2 and Table S7) (*vide infra*). These results position GAMA2 as a systematically improvable fragmentation scheme that complements existing FBQC methods for covalently connected biomolecules.

The accuracy of GAMA2 is primarily controlled by two parameters: the grid-box size and the cutoff radius used in the many-body expansion, which together determine the fragment size, overlap, and balance between cost and accuracy. To assess grid size dependence of the GAMA2 performance, we evaluated Segglu, β-strand-Ala_18_, and α-helix-Ala_18_ while fixing the cutoff radius at 5 Å and the low level at HF/6-311G­(d,p). As shown in Figure 3 (Table S3), increasing the grid-box size from 1 to 3 Å consistently improves accuracy, reflecting the ability of larger boxes to more effectively incorporate longer range fragment interactions and reduce many-body truncation error. We next examined the influence of the cutoff radius using Gly_12_ and α-helix-Ala_18_ with a fixed 2 Å grid box and a B3LYP/6-311G­(d,p) low-level method. As shown in Figure 4 (Table S4), increasing the cutoff radius from 5 to 9 Å systematically decreases GAMA2 errors, approaching a plateau once long-range electrostatics and higher order interactions are sufficiently captured. Beyond ∼5 Å, improvements are modest for both systems, supporting the use of a 5 Å cutoff radius as an optimal two-body truncation distance. A similar convergence behavior was also observed previously in our work for medium-sized water clusters.^7,8^

**Figure 3:**
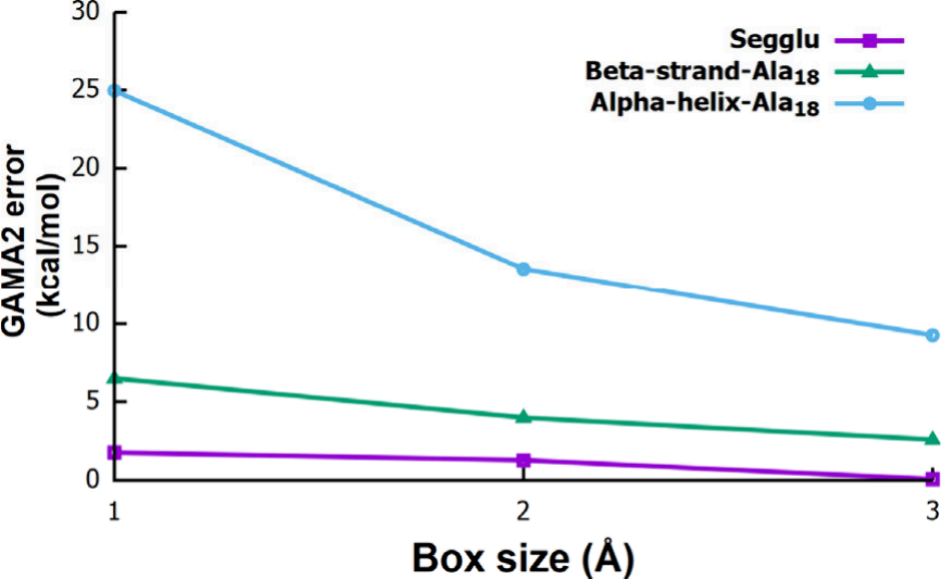
Effect of the grid-box size on absolute GAMA2 errors for three different peptide systems. The box size is varied from 1 to 3 Å, while the cutoff radius is fixed at 5 Å. All calculations use MP2/6-311G­(d,p) as the high-level method and HF/6-311G­(d,p) as the low-level method.

**Figure 4:**
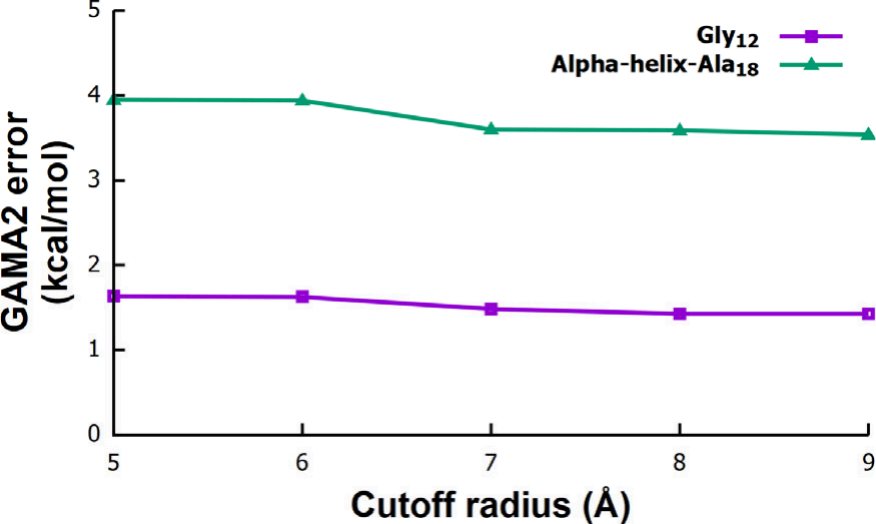
Effect of the cutoff radius on absolute GAMA2 errors for two different peptide systems. The cutoff radius is varied from 5 to 9 Å with a fixed grid-box size of 2 Å. All calculations use MP2/6-311G­(d,p) as the high-level method and B3LYP/6-311G­(d,p) as the low-level method.

We also performed a detailed evaluation of the computational timings (Figure 5) associated with GAMA2 and compared them to the corresponding wall-time requirements for full MP2 calculations. For this comparison, we selected two representative peptide systems: a medium-sized peptide (Gly_12_) and a larger α-helical peptide (Ala_18_). Across both systems, GAMA2 exhibits a substantial reduction in computational expense relative to that of full MP2, demonstrating its practical efficiency and scalability for biomolecular applications. The total computational time for GAMA2 is defined as the sum of three components: the full HF calculation time, the GAMA–HF correction time, and all of the GAMA–MP2 fragment-calculation times. In the GAMA2 workflow, the GAMA–HF and GAMA–MP2 steps are parallelized across 40 nodes, each equipped with 8 processors, enabling highly efficient execution of the fragment-based tasks. In contrast, the full HF and full MP2 calculations for both peptide systems were carried out on a single node with 8 processors. The α-helix-Ala_18_ system clearly highlights the magnitude of the computational advantage of GAMA2. The full MP2 calculation for this peptide requires approximately 8 days (192 h) of wall time, whereas the corresponding GAMA2 calculation completes in about 1.17 h, representing an improvement of nearly 164-fold. A similar trend is observed for the Gly_12_ peptide, confirming that the computational gains offered by GAMA2 are consistent across different peptide sizes. These results are illustrated in Figure 5 and summarized in Table S5 and Table S6.

**Figure 5:**
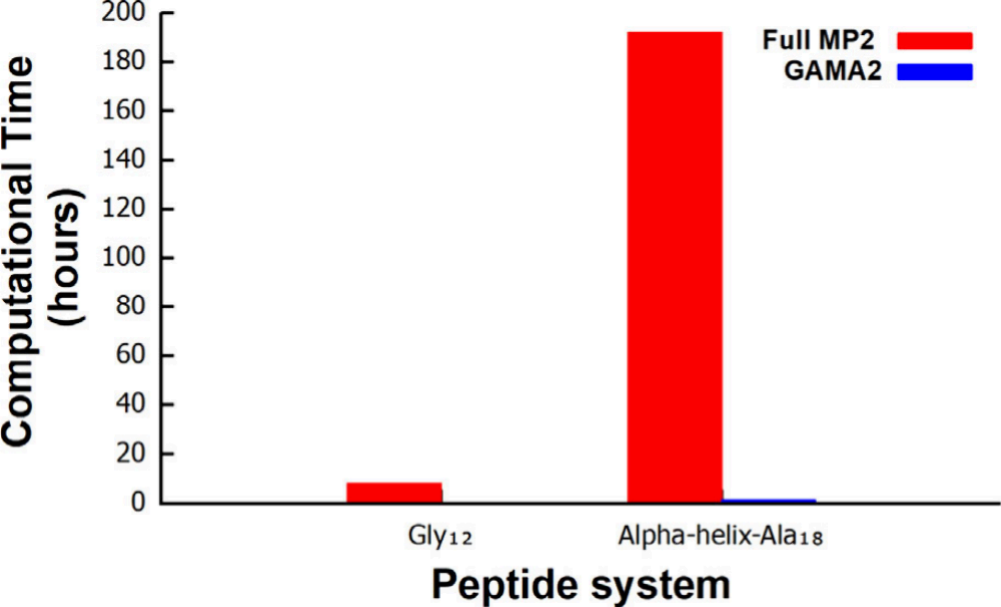
Wall-clock times (in hours) for a medium-sized peptide (Gly_12_) and a larger peptide (α-helix-Ala_18_) from full MP2 calculations versus the GAMA2 workflow. For GAMA2, the low-level correction uses HF/6-311G­(d,p), and the total time includes the full-system HF step, the GAMA–HF correction, and the GAMA–MP2 fragment calculations. In the bar plot, the GAMA2 timings (blue bars) are nearly invisible compared to those of full MP2, illustrating the substantial computational savings in the GAMA2 approach. Here, all GAMA calculations (both MA HF and GAMA MP2) were performed using a box size of 2 Å and a cutoff radius of 5 Å.

In addition to the wall-clock time, we also reported the corresponding CPU times for both the GAMA2 and full MP2 calculations. As shown in Figure S2 and Tables S7 and S8, a substantial reduction in computational cost is observed for GAMA2 relative to full MP2 when assessed in terms of CPU time. For example, the full MP2 calculation for the α-helix-Ala_18_ system requires approximately 1178 CPU hours, whereas the corresponding GAMA2 calculation requires only ∼77 CPU hours, corresponding to an approximately 15-fold reduction in CPU time. One important point to note is that, as shown in Table S8, the total CPU time for GAMA HF calculations is larger than that of the corresponding full HF calculations for both Gly_12_ and α-helix-Ala_18_. However, in terms of wall-clock time, GAMA HF consistently exhibits a lower computational cost than the full HF calculations. This behavior is fully consistent with prior literature.^14^ For example, Herbert and co-workers^14^ reported that fragment-based HF calculations can require substantially larger total CPU time than full HF calculations, while still yielding reduced wall-clock times due to efficient parallel execution (see Figure 9 of ref 14). Thus, it is possible for fragment-based HF calculations to exhibit a higher total CPU time but lower wall-clock time compared to full HF calculations. In contrast, GAMA MP2 calculations show a substantial reduction in both CPU and wall-clock times relative to full MP2 calculations. This behavior arises from the steep $O\left(N^{5}\right)$ scaling of supersystem MP2 calculations, which is dominated by electron-correlation contributions. Within GAMA, the single, computationally demanding full-system MP2 calculation is replaced by many significantly smaller fragment MP2 calculations involving far fewer basis functions. It is an important point to note that the formal computational scaling of GAMA calculations using MP2 can be expressed as $N_{F}\times O\left(n^{5}\right)$ , where *N*_F_ is the number of fragments and *n* denotes the size of the largest fragment. As a result, the computational cost is dramatically reduced. Furthermore, because the fragment MP2 calculations are independent, they can be efficiently parallelized, leading to pronounced reductions in both CPU and wall-clock times. We have also provided a detailed distribution of GAMA fragment counts as a function of the residue size for the two representative peptide systems, Gly_12_ and 3_10_-helix-Ala_18_, in Figure S3 (Table S9) and Figure S4 (Table S10), respectively.

In this work, conventional direct MP2 is employed for both the supersystem and GAMA2 fragment calculations to ensure a consistent and unbiased comparison. Under an ideal linear scaling scenario, the supersystem MP2 wall time for α-helix-Ala_18_ could be projected to ∼4.8 h on 40 nodes (i.e., 192 h divided by 40), corresponding to an approximately 5-fold speed-up as achieved by GAMA. In practice, however, parallel efficiency in canonical MP2 is limited by its formal $O\left(N^{5}\right)$ scaling, expensive integral transformations, internode communication overhead, and load imbalance. Linear-scaling variants of MP2, such as RI-MP2 ^22–25^ and local-MP2,^26^ significantly reduce computational cost but rely on additional approximations. RI-MP2 ^22,23^ replaces four-center integrals with density-fitted representations using an auxiliary basis set, introducing small but non-zero errors that depend on auxiliary basis quality. Local-MP2 ^26^ further exploits spatial locality by truncating weak orbital-pair correlations, which can reduce accuracy for extended or electronically delocalized systems where correlation effects are inherently non-local. To avoid conflating fragmentation errors with approximations from linear-scaling MP2 methods, we deliberately employed canonical MP2 as a common reference. Future work will incorporate RI-MP2 and local-MP2 consistently for both supersystem and fragment calculations to enable a balanced assessment of the computational efficiency and scalability within the GAMA2 framework.

In summary, we generalized the GAMA framework to enable robust, automated, and accurate fragment-based quantum chemistry for covalently bonded peptides. The GAMA2 protocol, employing a simple grid-based fragmentation and amany-body expansion with overlapping fragments truncated at two-body order with a multilayer correction, delivers correlated MP2-level energies for a diverse set of biomolecular structures with remarkable efficiency. Key to its performance is the systematic convergence of energy with respect to grid size and cutoff radius and the critical role of the low-level method, where DFT-based methods (B3LYP and M06-2X) provide good accuracy even for very larger and helical peptide systems. Specifically, GAMA2 is designed for applications where relative energetics, conformational preferences, and qualitative to semi-quantitative energetic trends are of primary interest, rather than sub-kcal mol^–1^ thermochemical accuracy. For flexible and moderately sized biomolecular systems, such as peptides and protein fragments, energy differences on the order of 1–4 kcal mol^–1^ are typically sufficient for reliable conformational ranking and structural analysis.^1,4,6,16^ For more compact and highly cooperative systems, errors of ∼2–5 kcal mol^–1^ still enable meaningful assessment of relative stability and energetic trends, particularly when full MP2 or higher level calculations are computationally prohibitive.^1,4,6,16^ In this context, GAMA2 provides a favorable balance between accuracy and computational efficiency, enabling correlated quantum-mechanical treatments of systems that are otherwise inaccessible with conventional supersystem methods. This combination of automation, accuracy, and computational efficiency makes GAMA2 a practical and systematically improvable framework for correlated wave function calculations on medium-sized biomolecular systems. More advanced electrostatically embedded variants, in which the full-system low-level correction is replaced by point-charge embedding, substantially reduce the computational cost and provide a pathway for extension of GAMA to larger biomolecular systems, which will be explored in future work.

## Supplementary Material





## Data Availability

The software tools used in this study are Gaussian 16 (http://www.gaussian.org/) and GaussView 6 (https://gaussian.com/gaussview6/). Fragmentation code (for example, Perl scripts) and input files of all the peptide systems are available for free on GitHub (https://github.com/SahaLabGitHub/GAMA-peptide).

## Notes

GAMA2 energy errors for all peptide systems of varying sizes considered in this work, analysis of the effect of three different low-level correction schemes on GAMA2 performance, influence of the box size on the accuracy of GAMA2, influence of the cutoff radius on the performance of GAMA2, detailed assessment of the computational cost of GAMA2 calculations and comparison to full MP2 calculations along with CPU time, analysis of the number of fragments with 1, 2, 3, and 4 amino acid residues, pictorial representations of all peptide systems examined in this study, future perspectives of the present work, and Cartesian coordinates of all peptide systems (PDF)
Transparent Peer Review report available (PDF)
