# Supplementary material for: GAMA: A Robust and Automated Fragment-Based Quantum Chemistry Method for Biomolecular Systems
Source: J Phys Chem Lett. 2026 Mar 18;17(13):3736–41. doi: 10.1021/acs.jpclett.5c03778 (PMC13051465; doi:10.1021/acs.jpclett.5c03778)
Supplement: Supplementary file 1 [file jz5c03778_si_001.pdf]

Supporting Information

GAMA: A Robust and Automated Fragment-Based Quantum Chemistry Method for Biomolecular Systems

Sujan Kundu, and Arjun Saha*

Department of Chemistry and Biochemistry, University of Wisconsin-Milwaukee

*Corresponding author; email: saha6@uwm.edu

Tables

Table S1. Absolute energy errors (kcal/mol) of GAMA2 (double-layer GAMA) with respect to reference Full MP2/6-311G(d,p) for different smaller to larger size peptides, with low-level correction using **HF/6-311G(d,p)** with fixed box size 2Å and fixed cutoff radius 5Å.

Peptide system	GAMA2 error (kcal/mol)
Segglu	1.29
Segtrp	0.01
Tuftsin	0.74
1YJP	0.52
Gly_{12}	3.87
β-strand-Ala_{18}	4.00
α-helix-Ala_{18}	13.53
3_{10}-helix-Ala_{18}	7.66

Table S2. Absolute energy errors (kcal/mol) of GAMA2 with respect to referenced Full MP2/6-311G(d,p) for different size medium and larger peptides with three different low-level corrections using box size 2Å and cutoff radius 5Å.

Peptide system	HF/6-311G(d,p)	B3LYP/6-311G(d,p)	M062X/6-311G(d,p)
1YJP	0.52	0.37	0.24
Gly_{12}	3.87	1.63	1.00
β-strand-Ala_{18}	4.00	2.80	1.50
α-helix-Ala_{18}	13.53	3.95	5.58
3_{10}-helix-Ala_{18}	7.66	4.40	2.70

Table S3. Absolute energy errors(kcal/mol) of GAMA2 with respect to Full MP2/6-311G(d,p) for one smaller peptide (Segglu) and two larger peptides (β-strand-Ala_{18}, α-helix-Ala_{18}) with low-level correction using **HF/6-311G(d,p)** with different box sizes and fixed cutoff radius 5Å.

Peptide system	B=1Å, R=5Å	B=2Å, R=5Å	B=3Å, R=5Å
Segglu	1.77	1.29	0.07
β-strand-Ala_{18}	6.50	4.00	2.59
α-helix-Ala_{18}	24.97	13.53	9.24

Table S4 : Absolute energy error (kcal/mol) of GAMA2 with respect to super system MP2/6-311g(d,p) for one representative large peptide system(α-helix-Ala_{18}) and one medium size peptide system (Gly_{12}) with fixed box size (box size = 2Å) and different cutoff radius with low level correction using **B3LYP/6-311G(d,p)**.

Peptide system	B=2Å, R=5Å	B=2Å, R=6Å	B=2Å, R=7Å	B=2Å, R=8Å	B=2Å, R=9Å
Gly_{12}	1.63	1.63	1.48	1.42	1.42
α-helix-Ala_{18}	3.95	3.94	3.60	3.59	3.54

Table S5. Computational **wall** times (in hours) for a medium-sized peptide (Gly_{12}) and a larger peptide (α-helix-Ala_{18}) obtained from full MP2 calculations and from the GAMA2 approach. All reported timings correspond to wall-clock time. Low-level corrections in GAMA2 were carried out using HF/6-311G(d,p). Here GAMA2 computational time is the sum of Full HF time , GAMA HF time and GAMA MP2 time.

Peptide system	Full MP2	GAMA2
Gly_{12}	8	0.23
α-helix-Ala_{18}	192	1.17

Table S6. Computational **wall** times (in hours) for a medium-sized peptide (Gly_{12}) and a larger peptide (α-helix-Ala_{18}) for Full HF calculations, GAMA HF calculations, GAMA MP2 calculations. All calculations were performed using 6-311G(d,p) basis set.

Peptide system	Full HF	GAMA HF	GAMA MP2
Gly_{12}	0.08	0.028	0.12
α-helix-Ala_{18}	0.58	0.025	0.56

Table S7. Computational **CPU** times (in hours) for a medium-sized peptide (Gly_{12}) and a larger peptide (α-helix-Ala_{18}) obtained from full MP2 calculations and from the GAMA2 approach. All reported timings correspond to **CPU** time. Low-level corrections in GAMA2 were carried out using HF/6-311G(d,p). Here GAMA2 computational time is the sum of Full HF time, GAMA HF time and GAMA MP2 time. All GAMA calculations (both GAMA HF and GAMA MP2) were performed using box size 2Å and cutoff radius 5Å.

**Peptide system**	**Full MP2**	**GAMA2**
Gly_{12}	61.74	51.68
α-helix-Ala_{18}	1178.00	77.49

Table S8. Computational **CPU** times (in hours) for a medium-sized (Gly_{12}) peptide and a larger peptide (α-helix-Ala_{18}) for Full HF calculations, GAMA HF calculations and GAMA MP2 calculations. All calculations were performed using 6-311G(d,p) basis set.

**Peptide system**	**Full HF**	**GAMA HF**	**GAMA MP2**
Gly_{12}	0.72	14.62	36.34
α-helix-Ala_{18}	3.49	16.00	58.00

Table S9. Distribution of GAMA fragment counts by residue size for the Gly_{12} peptide obtained using a box size of 2 Å and a cutoff radius of 5 Å (total fragments = 84). The largest fragment contains four amino acid residues, while the smallest fragment contains a single residue. Fragments are generated by breaking the bond between α carbon and carbonyl carbon across the peptide backbone.

**Fragment size**	**Number of fragments**
1 amino acid residues	13
2 amino acid residues	17
3 amino acid residues	32
4 amino acid residues	22

Table S10. Distribution of GAMA fragment counts by residue size for the 3_{10}-helix-Ala_{18} peptide obtained using a box size of 2 Å and a cutoff radius of 5 Å (total fragments = 104). The largest fragment contains four amino acid residues, while the smallest fragment contains a single residue. Fragments are generated by breaking the bond between α carbon and carbonyl carbon across the peptide backbone.

**Fragment size**	**Number of fragments**
1 amino acid residues	18
2 amino acid residues	18
3 amino acid residues	37

4 amino acid residues	31

Figures

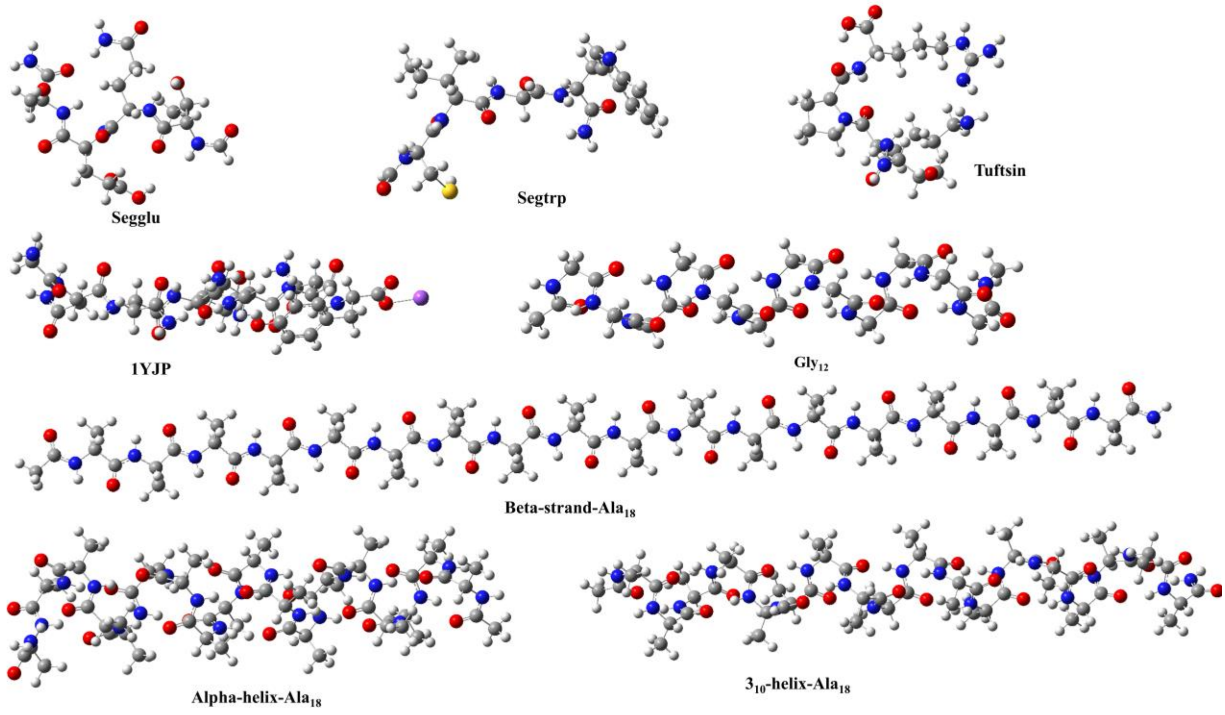

Figure S1. Pictorial representation of the peptide systems considered in this work. Grey, white, red, blue, and yellow spheres represent carbon, hydrogen, oxygen, nitrogen, and sulfur atoms, respectively.

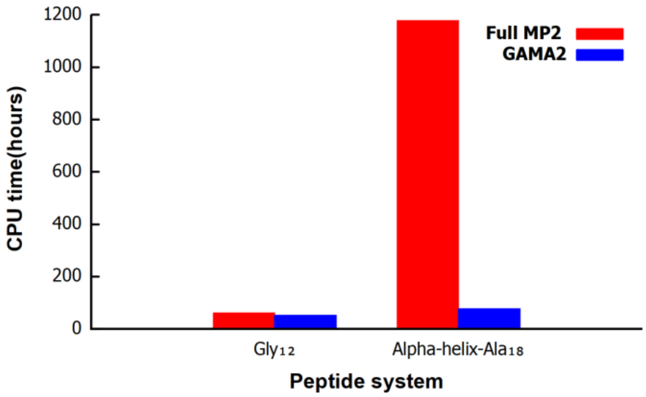

Figure S2. CPU times (in hours) for a medium-sized peptide (Gly_{12}) and a larger peptide (α-helix-Ala_{18}) from full MP2 calculations versus the GAMA2 workflow. For GAMA2, the low-level correction uses HF/6-311G(d,p), and the total time includes the full-system HF step, the GAMA–HF correction, and the GAMA–MP2 fragment calculations. All GAMA calculations

(both GAMA HF and GAMA MP2) were performed using box size 2Å and cutoff radius 5Å. **Table S7** contains corresponding numbers.

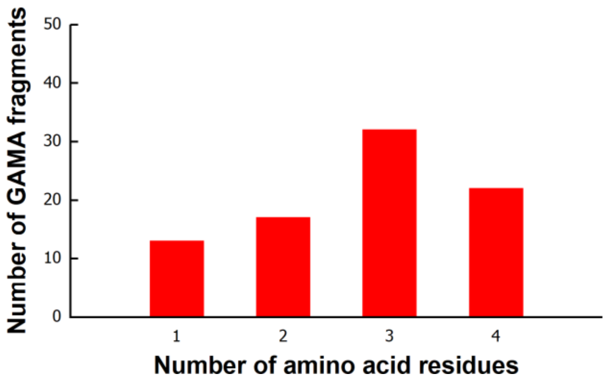

Figure S3. Histogram plot of the distribution of GAMA fragment counts by residue size for the Gly_{12} peptide obtained using a box size of 2 Å and a cutoff radius of 5 Å (total fragments = 84). The largest fragment contains four amino acid residues, while the smallest fragment contains a single residue. **Table S9** contains corresponding numbers.

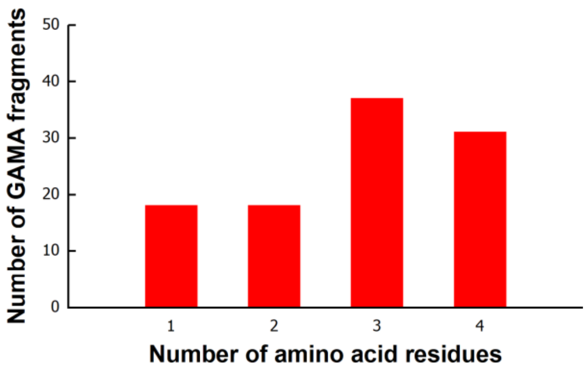

Figure S4. Histogram plot of the distribution of GAMA fragment counts by residue size for the 3_{10}-helix-Ala_{18} peptide obtained using a box size of 2 Å and a cutoff radius of 5 Å (total fragments = 104). The largest fragment contains four amino acid residues, while the smallest fragment contains a single residue. **Table S10** contains corresponding numbers.

<u>Future Perspective of the present work</u>

In the present work, GAMA has been applied and validated only for medium-sized peptide systems, with a focus on evaluating total energies. While this represents an important proof-of-concept, the framework is inherently flexible and holds significant potential for extension to larger and more complex biomolecular systems. One promising direction is the integration of electrostatically embedded variant, EE-GAMA[1], which we have introduced in our prior work in which the full-system low-level correction can be replaced by point charges. This

modification allows the treatment of very large systems, such as protein–protein and protein–ligand complexes, while retaining the systematically improvable nature of the fragmentation protocol. In addition, we plan to explore another variant of GAMA in future in which strongly overlapping fragments will be generated by merging adjacent grid boxes. This approach further reduces reliance on low-level HF-based corrections and expands the applicability of GAMA to much larger biomolecular assemblies. From a biological perspective, GAMA and its variants are particularly suited for applications where accurate total or relative energies are critical. Potential applications include comparing the relative stabilities of peptide or protein conformations, evaluating the energetic effects of localized mutations, assessing relative binding or association energies of biomolecular fragments, and benchmarking correlated wavefunction methods against DFT for biologically relevant subsystems. Furthermore, GAMA-based approaches could be extended to predict protein–ligand and protein–protein binding affinities, providing a valuable tool for drug discovery and rational biomolecular design. The systematic and modular nature of GAMA makes it a flexible platform for future methodological developments, including the calculation of energy gradients, geometry optimizations, and interaction energies, which are beyond the scope of the present work but represent natural extensions. The implementation and thorough validation of these capabilities will be addressed in future studies, highlighting the potential of GAMA as a general, systematically improvable framework for studying increasingly complex biomolecular systems efficiently and accurately.

There are several strategies, which we are also planning to implement in our future work to reduce or avoid full-system HF calculations in GAMA protocol. For instance, one approach is "forced overlapping fragment generation" through systematic merging of adjacent grids followed by the application of MOBE. Another way is to combine this grid merging protocol with electrostatic charge embedding variant of GAMA[1]. Further strategies under consideration include:

1. **Layered Theoretical Models based on Interaction Proximity:** Within a fixed n-body expansion, the level of theory decreases as the interaction cutoff radius increases. For instance, with a desired 6 Å total interaction cutoff radius, interactions can be treated hierarchically: CCSD(T) for fragments within 2 Å, MP2 up to 3 Å, B3LYP up to 4 Å, HF up to 5 Å, and semi-empirical PM6 for the remaining fragments up to 6 Å.

2. **Multi-level Treatment of n-body Interactions:** Within a fixed interaction cutoff, the level of theory is scaled inversely with the order of the n-body expansion. For example, at a 5 Å cutoff, 2-body interactions are treated with MP2, while 3-body and 4-body terms are assigned to the more computationally efficient HF and PM6 methods, respectively.

These advanced approaches promise to capture medium- to long-range interactions efficiently, reducing dependence on a single low-level theory. Consequently, GAMA could potentially be applied to extremely large biomolecular systems, including those exceeding 10,000 atoms. Finally, we are exploring integration with machine learning approaches to address persistent challenges in FBQC, such as redundant fragment calculations, accurate treatment of long-range inter-fragment interactions and to develop efficient and user-friendly fragmentation protocols. Together, these strategies highlight the long-term potential of GAMA as a systematically improvable and widely applicable framework for large-scale, accurate quantum chemical calculations on biomolecular systems.

<u>Coordinates of all peptide systems considered in this study</u>

Segglu

```
C 5.32199300 -2.37494700 0.50223200
O 6.08199300 -1.88894700 -0.35176800
N 3.98899300 -2.29094700 0.42323200
C 3.41899300 -1.57294700 -0.73376800
C 2.25499300 -0.67494700 -0.33976800
O 2.07599300 -0.28094700 0.82123200
C 3.14999300 -2.54594700 -1.89176800
O 2.69399300 -1.88894700 -3.09176800
C 2.12799300 -3.61894700 -1.52576800
N 1.43899300 -0.31494700 -1.31176800
C 0.46299300 0.74705300 -1.23876800
C -0.59900700 0.71205300 -0.17876800
O -0.88800700 1.74405300 0.45623200
C -0.20800700 0.90805300 -2.64176800
C 0.85599300 1.33405300 -3.66076800
C 0.35699300 1.53305300 -5.07276800
O 1.15299300 1.54705300 -6.02376800
N -0.95200700 1.69905300 -5.24876800
N -1.26200700 -0.41094700 0.03423200
C -2.40100700 -0.51894700 0.95123200
C -3.62800700 0.14405300 0.33423200
O -4.61900700 0.46405300 1.01923200
C -2.17800700 -0.05794700 2.39523200
C -0.84800700 -0.40494700 3.04923200
C -0.50300700 -1.87694700 3.12623200
O 0.57099300 -2.18794700 3.69623200
O -1.27500700 -2.72494700 2.62423200
```

N -3.62900700 0.31205300 -0.99176800
C -4.80400700 0.79605300 -1.71176800
C -4.63700700 0.39705300 -3.18076800
O -3.60100700 -0.14994700 -3.55876800
C -5.16600700 2.25005300 -1.53376800
O -4.23100700 3.13605300 -2.10976800
N -5.67000700 0.60005300 -3.98676800
H 4.10083500 -2.99865600 -2.08111900
H 1.80132200 -3.47128400 -0.51761000
H 2.57753900 -4.58562900 -1.61706000
H 1.28868100 -3.55211700 -2.18606200
H 2.81510600 -0.94074500 -3.00320400
H 4.14246900 -0.88081800 -1.11118000
H 3.42662500 -2.70153300 1.14098000
H -0.90320800 1.71753900 -2.56221700
H -0.54854900 -0.06266200 -2.93612200
H 1.19194800 2.29722100 -3.33775000
H 1.54493700 0.51705400 -3.71338400
H -1.57155300 1.65402400 -4.46510000
H -1.31895800 1.84012000 -6.16825000
H 1.05215600 1.58514400 -0.92991400
H 1.72466700 -0.67120300 -2.20141500
H -2.91399500 -0.58059300 2.96974400
H -2.19423200 1.01099300 2.35047200
H -0.94047900 -0.08300500 4.06545200
H -0.10099900 0.02751200 2.41688900
H -2.55426000 -1.57178000 1.06506000
H -1.02545200 -1.23620300 -0.47859800
H -5.13390800 2.43368100 -0.48013100
H -6.07816900 2.39375400 -2.07433200
H -4.45314300 4.03790400 -1.86703400
H -5.65745300 0.32516400 -1.27041500
H -2.82725600 0.05107500 -1.52943500
H -6.49399700 1.04837900 -3.64029500
H 5.73247500 -2.94032700 1.31263400
H -5.63726000 0.25143700 -4.92346100
H 1.31311193 -1.89626880 3.16164494

Segtrp

C -4.32256800 -4.09237900 -3.57998200
O -3.80356800 -4.54137900 -4.61198200
N -3.68456800 -4.02237900 -2.41898200
C -2.32056800 -4.53437900 -2.25198200
C -1.94356800 -4.42937900 -0.77498200
O -2.64456800 -3.72437900 -0.03598200
C -1.29756800 -3.87837900 -3.15598200
S -1.33956800 -2.10137900 -3.38098200
N -0.85056800 -5.03637900 -0.35798200
C -0.45256800 -5.02837900 1.05801800
C 0.67543200 -4.03737900 1.30101800
O 1.63943200 -4.03137900 0.53001800
C 0.03743200 -6.43837900 1.45301800
C -1.02856800 -7.50837900 1.13901800
C 0.46443200 -6.51237900 2.92401800
C -2.36356800 -7.25837900 1.80201800
N 0.54443200 -3.22737900 2.36101800
C 1.61343200 -2.25137900 2.59801800

C 1.14743200 -1.11937900 3.49301800
O -0.02556800 -1.08637900 3.89001800
N 2.05543200 -0.19137900 3.79201800
C 1.76843200 0.90862100 4.70401800
C 1.99943200 2.28662100 4.10501800
O 2.01443200 3.29562100 4.81001800
C 2.56743200 0.71462100 6.00801800
C 3.98243200 0.23662100 5.83401800
C 4.46843200 -0.97337900 6.25301800
C 5.08443200 0.92962100 5.23601800
N 5.81043200 -1.06437900 5.94401800
C 6.20743200 0.08862100 5.32301800
C 5.24143200 2.18662100 4.64201800
C 7.46543200 0.44062100 4.83301800
C 6.47843200 2.54162100 4.12901800
C 7.58643200 1.67362100 4.22901800
N 2.10243200 2.36962100 2.78201800
H -0.35688600 -4.06900800 -2.68303600
H -1.49879400 -4.27890200 -4.12757400
H -2.22938200 -1.57986900 -2.57329600
H -2.31096300 -5.55900500 -2.56012400
H -4.14891600 -3.65117900 -1.61487400
H 0.90414200 -6.64179000 0.85943700
H 0.33260800 -5.55451400 3.38229200
H -0.13556100 -7.23714800 3.43354500
H 1.49418800 -6.79711000 2.98260000
H -0.65973600 -8.41988800 1.56093600
H -1.20857800 -7.44040000 0.08646100
H -2.31196700 -6.35939500 2.38000100
H -2.60320400 -8.08111100 2.44278800
H -3.12065800 -7.15764200 1.05264000
H -1.29944000 -4.74149700 1.64573000
H -0.31646600 -5.59575300 -0.99188800
H 2.37769300 -2.76813700 3.14002000
H 1.83533000 -1.81180300 1.64805200
H -0.26737600 -3.29084200 2.94148300
H 2.06471500 -0.06767100 6.53735200
H 2.64655600 1.68934800 6.44223700
H 3.89556200 -1.73176700 6.74451300
H 6.30510000 -1.11124900 6.81183500
H 4.41628200 2.86539700 4.58466000
H 6.59565700 3.49050500 3.64862500
H 8.30244400 -0.21991300 4.92250200
H 8.53237000 1.97639300 3.83098400
H 0.71976600 0.87735400 4.91430500
H 2.98822900 -0.27314100 3.44101200
H 2.08822900 1.53839000 2.22627100
H -5.32432600 -3.71672000 -3.59616200
H 2.17702500 3.21751700 2.25712900

Tuftsin

C 1.30470400 1.42712500 1.49669200
O 0.34492000 1.47398900 2.23189000
N 1.58795500 2.44582800 0.65566600
C -0.47869700 3.40144000 -0.22696300
O -0.52281000 3.75783600 -1.38489400
N -1.48342500 2.73171200 0.35359300

C -3.65217700 3.09267600 -0.75431800
C -3.83092600 -3.27629600 -1.27347100
N -2.59163400 -3.22890700 -1.01209600
O -4.77396600 2.95242400 -0.39604300
H -1.31446800 2.37643000 1.27030300
H -2.08818300 -3.95606000 -1.48256700
N -4.73056500 -2.44239500 -0.66081400
H -5.54424100 -2.24755300 -1.20075600
C -4.27089900 -1.35253900 0.17903400
H -5.09691700 -1.06277000 0.82039600
H -3.48757900 -1.74314300 0.81261000
C -3.76281100 -0.13991000 -0.60227500
H -4.59336000 0.34053000 -1.11080000
H -3.06450500 -0.48707200 -1.35719000
C -3.05835100 0.86041600 0.31323900
H -3.72122100 1.18175900 1.10960700
H -2.20737400 0.36523500 0.77186400
C -2.53791900 2.09028300 -0.42906700
H -2.10180100 1.78821300 -1.37719600
O -3.28345400 4.12588100 -1.48642500
H -2.35190800 4.11030300 -1.69216100
C 0.74786200 3.65214700 0.65869500
H 0.41953400 3.84637900 1.67090900
C 1.65819500 4.76717700 0.11504400
H 1.37536500 4.99615300 -0.90259500
H 1.56296100 5.67171200 0.70274800
C 3.06980100 4.17428900 0.15990700
H 3.73079700 4.61276100 -0.57814900
H 3.51776400 4.31455000 1.13898900
C 2.83042900 2.68773200 -0.08007600
H 2.68035800 2.48414300 -1.13639600
H 3.63509200 2.06433100 0.27295000
C 2.23029700 0.20111300 1.49382400
H 3.22507200 0.54392400 1.74280600
C 1.82940600 -0.84614300 2.54337100
H 2.66218100 -1.54157300 2.61409500
H 1.75457700 -0.34476700 3.50231100
C 0.54132800 -1.63140600 2.27908500
H 0.59370600 -2.12127200 1.31057000
H -0.30034400 -0.94826600 2.25769200
C 0.30401400 -2.70370300 3.34460400
H 0.30796800 -2.23733800 4.32879400
H 1.12065800 -3.42119400 3.32517100
C -1.01003500 -3.46321200 3.17548000
H -1.09146100 -4.18119300 3.98622000
H -1.84406100 -2.76630600 3.29603000
N -1.05979600 -4.19783000 1.92012800
H -1.75216500 -4.91779500 1.96764100
H -1.32541200 -3.61069400 1.15346400
N 2.32739000 -0.35549100 0.15461700
H 1.50439600 -0.74366400 -0.24538000
C 3.50824700 -0.62442300 -0.44679100
O 4.56913600 -0.28035500 0.01012400
C 3.44409600 -1.37036400 -1.77803600
H 2.42770700 -1.38673700 -2.15925900
C 3.90724000 -2.83271200 -1.62304500
H 4.93441700 -2.80811400 -1.25863200
N 4.29250400 -0.74027900 -2.77637500

H 3.87222200 0.08623600 -3.15075600
H 5.16407000 -0.47799800 -2.35729400
O 3.84986500 -3.45110400 -2.87335000
H 4.24607600 -2.86344300 -3.50066000
C 3.05955600 -3.65474000 -0.66799700
H 3.12949200 -3.28912100 0.35058300
H 2.01770500 -3.65009500 -0.97323200
H 3.40359900 -4.68163900 -0.68116400
N -4.43587400 -4.12486300 -2.18486100
H -3.86226800 -4.86581800 -2.51891300
H -5.35403800 -4.42652800 -1.94368700

1YJP

N -9.00900000 4.61200000 6.10200000
C -9.05200000 4.20700000 4.65100000
C -8.01500000 3.14000000 4.41900000
O -7.52300000 2.52100000 5.38100000
N -7.65600000 2.92300000 3.15500000
C -6.52200000 2.03800000 2.83100000
C -5.24100000 2.53700000 3.42700000
O -4.97800000 3.74200000 3.42600000
C -6.34600000 1.88100000 1.34100000
C -7.58400000 1.34200000 0.69200000
O -8.02500000 0.22700000 1.01600000
N -8.20400000 2.15500000 -0.16900000
N -4.43800000 1.59000000 3.90500000
C -3.19300000 1.90400000 4.58900000
C -1.95500000 1.33200000 3.89500000
O -1.87200000 0.11900000 3.64800000
C -3.25900000 1.37800000 6.04200000
C -2.00600000 1.73900000 6.86100000
O -1.70200000 2.92500000 7.07200000
N -1.27100000 0.71500000 7.30600000
N -1.00500000 2.22800000 3.59800000
C 0.38400000 1.88800000 3.19900000
C 1.43500000 2.60600000 4.08800000
O 1.54700000 3.84300000 4.11500000
C 0.65600000 2.14800000 1.71100000
C 1.94400000 1.45800000 1.21300000
C 2.50400000 2.04400000 -0.08900000
O 2.74400000 3.26800000 -0.19000000
N 2.75000000 1.16100000 -1.09100000
N 2.15400000 1.82100000 4.87100000
C 3.27000000 2.36100000 5.64000000
C 4.59400000 1.76800000 5.17200000
O 4.76800000 0.54600000 5.05400000
C 3.05600000 2.18300000 7.14700000
C 1.82900000 2.95000000 7.64700000
C 1.34400000 2.41400000 8.95400000
O 0.77400000 1.32500000 9.00200000
N 1.54900000 3.18700000 10.03900000
N 5.51400000 2.66400000 4.85600000
C 6.83100000 2.31000000 4.31800000
C 7.85400000 2.76100000 5.32400000
O 8.21900000 3.94300000 5.37400000
C 7.06500000 3.01600000 2.99300000
C 5.96100000 2.73500000 2.00300000

O 5.79800000 1.60400000 1.55100000
N 5.19500000 3.74700000 1.67900000
N 8.29200000 1.81700000 6.14700000
C 9.15900000 2.14400000 7.29900000
C 10.60300000 2.33100000 6.88500000
O 11.04100000 1.81100000 5.85500000
C 9.06100000 1.06500000 8.36900000
C 7.66500000 0.92900000 8.90200000
C 6.77100000 0.02100000 8.32700000
C 7.21000000 1.75600000 9.92000000
C 5.48000000 -0.09400000 8.79600000
C 5.90400000 1.64900000 10.41600000
C 5.04700000 0.72900000 9.83100000
O 3.76600000 0.58900000 10.29100000
O 11.35800000 2.99900000 7.61200000
H -10.00732562 3.75607410 4.48094474
H -8.75424756 5.06120173 4.07952568
H -9.89861124 4.43637120 6.52359978
H -8.79448663 5.58635823 6.16989743
H -5.58758591 1.13864831 1.20453744
H -6.20625436 2.86689362 0.94935612
H -7.68481456 3.00504720 -0.25769185
H -9.12298437 2.35933934 0.16821383
H -6.75490866 1.08439194 3.25677602
H -7.37039377 3.82165248 2.82205435
H -4.07496864 1.89119347 6.50646490
H -3.26940864 0.31042216 5.97080162
H -0.46805040 1.06566103 7.78798421
H -1.81840388 0.15862170 7.93113373
H -3.09059895 2.96893647 4.57099027
H -4.98985109 1.13492420 4.60383217
H 0.82896095 3.20003467 1.62040441
H -0.15090382 1.69058528 1.17754146
H 2.68482091 1.64974297 1.96087633
H 1.66448149 0.45118570 0.98257784
H 2.47489877 0.24398602 -0.80220134
H 3.72670840 1.16391989 -1.30555109
H 0.48366614 0.83425888 3.35583210
H -0.88391541 2.72998160 4.45436032
H 3.90301823 2.62989591 7.62422548
H 2.84231087 1.14552556 7.29827372
H 2.15575863 3.95143082 7.83479008
H 1.04914386 2.76579634 6.93792073
H 1.15088052 2.74530818 10.84299578
H 2.53133102 3.31115657 10.17903895
H 3.31338299 3.41491027 5.46030290
H 1.50703896 1.50212211 5.56364587
H 7.02148863 4.06477159 3.20056905
H 7.95448931 2.58900944 2.57900987
H 5.51940775 4.57210345 2.14156232
H 4.25418391 3.55596945 1.95895074
H 6.89821213 1.25600366 4.14631932
H 5.72467458 3.10569995 5.72807647
H 9.66281476 1.39988800 9.18788279
H 9.28714403 0.13931892 7.88230357
H 7.09320707 -0.59286643 7.51198429
H 7.86973589 2.48964116 10.33402802
H 4.81396490 -0.81273458 8.36621186

H 5.57524051 2.26339742 11.22799323
H 3.62857860 1.17534727 11.03860433
H 8.81015750 3.07186907 7.70182490
H 7.46025730 1.44922990 6.56287165
Na 13.03645064 2.12019087 6.40128780

Gly_{12}

C -12.87035289 0.56179639 2.16179006
C -11.71952495 -0.07286062 1.42045607
O -10.54898977 0.03899150 1.75984838
H -12.47781856 1.18084623 2.95258624
H -13.50145296 -0.20794566 2.59206961
H -13.47297864 1.16452709 1.49238548
N -12.05989879 -0.78980221 0.31555836
C -11.06857409 -1.63456466 -0.33979848
C -9.86646379 -0.86405869 -0.85949491
O -8.77214321 -1.40465154 -0.95771807
H -13.02222373 -0.92242090 0.08920359
H -10.67500645 -2.38199920 0.33356612
H -11.54102541 -2.13406968 -1.17523325
N -10.07756006 0.41412503 -1.21263986
C -8.98014867 1.23020758 -1.70239489
C -7.83066778 1.36521790 -0.71111020
O -6.71459598 1.67996118 -1.11871007
H -10.99086413 0.80146896 -1.13441515
H -8.54968096 0.81228121 -2.60019040
H -9.35447733 2.21923788 -1.92857601
N -8.09310509 1.12141800 0.57659695
C -7.01438858 1.16107165 1.54680412
C -5.89959385 0.16838229 1.24614902
O -4.77235555 0.35023504 1.70323968
H -9.00148103 0.84161791 0.89558601
H -6.54585756 2.13310507 1.58142862
H -7.42542326 0.93531971 2.52115636
N -6.20365900 -0.87725053 0.46732772
C -5.16087292 -1.80924500 0.08289565
C -4.01653982 -1.15254705 -0.67806841
O -2.91200338 -1.69311299 -0.71623389
H -7.12581477 -1.01749873 0.09945081
H -4.71142531 -2.28220529 0.94327084
H -5.59988837 -2.57216037 -0.54528163
N -4.26871513 0.01760285 -1.27622590
C -3.19230585 0.72841646 -1.93845646
C -2.03810756 1.07591357 -1.00757964
O -0.92005413 1.30218345 -1.47006047
H -5.16838784 0.45932402 -1.23055465
H -2.76186495 0.14564095 -2.73908972
H -3.59223362 1.64543904 -2.34896784
N -2.29712282 1.10878804 0.30446363
C -1.21631718 1.34616295 1.24075304
C -0.10449660 0.30940476 1.15303970
O 1.02218873 0.57755451 1.57010695
H -3.20863686 0.91275706 0.67673944
H -0.74361837 2.30213811 1.07166209

H -1.62454158 1.32982419 2.24200510
N -0.40836758 -0.87128780 0.60206287
C 0.63519788 -1.85905946 0.41025526
C 1.77236030 -1.37046950 -0.47777141
O 2.87717308 -1.90981542 -0.41740322
H -1.32699062 -1.08140456 0.25510145
H 1.09249992 -2.14406659 1.34601707
H 0.19396303 -2.73503927 -0.04513710
N 1.51279672 -0.34362451 -1.29519967
C 2.58257873 0.22399946 -2.09184215
C 3.73637095 0.76614165 -1.25779260
O 4.84727183 0.91139041 -1.76611925
H 0.61192642 0.09827172 -1.33251992
H 3.01681310 -0.50763577 -2.75677169
H 2.17380300 1.03352838 -2.68086654
N 3.48260830 1.05648523 0.02325257
C 4.56462925 1.48683958 0.88677359
C 5.67959101 0.45750421 1.02244455
O 6.79678204 0.80672226 1.39577950
H 2.57571592 0.92609497 0.43360281
H 5.03610770 2.38544453 0.51770168
H 4.15700179 1.68323426 1.86892687
N 5.38324182 -0.81135624 0.71240476
C 6.43265631 -1.81054568 0.73290557
C 7.56115844 -1.53466827 -0.25485447
O 8.65035571 -2.08659955 -0.10822732
H 4.47018851 -1.08982359 0.40280304
H 6.90090871 -1.87440698 1.70392579
H 5.99444521 -2.76923823 0.49166239
N 7.30419326 -0.68321433 -1.25400224
C 8.36010145 -0.31168003 -2.17422215
C 9.50032634 0.49271304 -1.55091093
O 10.52841333 0.66278965 -2.18890991
H 6.41115262 -0.23888007 -1.36023814
H 8.82246733 -1.18502162 -2.61038271
H 7.92715084 0.28325847 -2.96615004
N 9.29464022 0.98323469 -0.31385562
C 10.36977293 1.64054362 0.40066375
C 11.12263842 0.74460275 1.38230406
O 11.96532476 1.23424210 2.12564777
H 8.41651759 0.87903550 0.15758279
H 11.09099433 2.00064070 -0.31816695
H 9.99122852 2.48111390 0.96432266
N 10.82291707 -0.55919795 1.36453736
C 11.52805758 -1.47693785 2.25102093
H 10.12729930 -0.93598698 0.75359916
H 11.50831729 -1.10999775 3.26871007
H 11.03750513 -2.43852565 2.20164817
H 12.56511068 -1.59050344 1.95692459

β-strand-Ala$_{18}$

C -29.09345000 -0.47393000 0.20327600
O -29.28242100 -1.48399400 0.88879700
N -27.87173800 -0.07527600 -0.22117700
H -27.73657400 0.75496600 -0.79394000
C -26.63213000 -0.76680300 0.13535800
C -25.47836600 0.18133600 -0.28804400

O -25.69462800 1.18327300 -0.97783800
N -24.25169600 -0.17884100 0.15054000
H -24.09551500 -1.00685900 0.72211400
C -23.02702500 0.54642500 -0.19310200
C -21.85545400 -0.37109300 0.25047300
O -22.06012000 -1.38074000 0.93312900
N -20.63131800 0.02222300 -0.16321600
H -20.48497700 0.85672900 -0.72837100
C -19.39435100 -0.67332800 0.19868800
C -18.23975900 0.27381500 -0.22783300
O -18.46193400 1.28216600 -0.90698200
N -17.01160200 -0.09323100 0.19665100
H -16.85135600 -0.92926200 0.75600200
C -15.78606300 0.62996400 -0.15018200
C -14.61689100 -0.29797600 0.27953900
O -14.82659800 -1.31679400 0.94716200
N -13.39205300 0.09618000 -0.12912100
H -13.24168600 0.94019700 -0.67924700
C -12.15696000 -0.60931000 0.22074000
C -11.00036200 0.34150400 -0.19290300
O -11.22274500 1.36396800 -0.85075100
N -9.77196200 -0.03819100 0.21837800
H -9.61152000 -0.88750100 0.75752000
C -8.54485200 0.68679200 -0.11962200
C -7.37822100 -0.25603600 0.28444600
O -7.59110700 -1.29047400 0.92666900
N -6.15281700 0.14302800 -0.11701500
H -5.99946400 1.00149700 -0.64355800
C -4.91981200 -0.57605000 0.21229800
C -3.76054100 0.38222900 -0.17620500
O -3.98105700 1.42485500 -0.80234600
N -2.53257000 -0.01398200 0.22012400
H -2.37328600 -0.88109300 0.73056800
C -1.30365200 0.71631500 -0.09962400
C -0.13965400 -0.24421600 0.26899100
O -0.35518400 -1.29945900 0.87552400
N 1.08636500 0.16362500 -0.12144700
H 1.24243100 1.04043100 -0.61601700
C 2.31720500 -0.57104300 0.18058000
C 3.47931300 0.39740200 -0.17269300
O 3.26158300 1.46389200 -0.75831600
N 4.70644000 -0.01745400 0.20679000
H 4.86390100 -0.90466400 0.68201400
C 5.93717600 0.72069800 -0.08700100
C 7.09865800 -0.25755100 0.24057700
O 6.88087500 -1.33548800 0.80489900
N 8.32536700 0.16146400 -0.13574800
H 8.48371700 1.05730800 -0.59408500
C 9.55415000 -0.58850300 0.13548500
C 10.71897900 0.39016000 -0.17820400
O 10.50453300 1.47996900 -0.72042500
N 11.94501800 -0.04292900 0.18437700
H 12.10006000 -0.94875900 0.62380300
C 13.17743000 0.70353600 -0.07985300
C 14.33678000 -0.28945200 0.20798900
O 14.11738400 -1.38794600 0.73034600
N 15.56430200 0.14098600 -0.15308400
H 15.72413000 1.05309600 -0.57738700

C 16.79114500 -0.62201500 0.08871600
C 17.95862900 0.36462500 -0.18692300
O 17.74835000 1.47368300 -0.69004400
N 19.18331000 -0.08415100 0.16202300
H 19.33493400 -1.00441500 0.57128800
C 20.41703600 0.66970900 -0.07304500
C 21.57505200 -0.33335500 0.18155900
O 21.35581500 -1.44781400 0.66848000
N 22.80346400 0.10723400 -0.16594200
H 22.96326900 1.03106400 -0.56353300
C 24.02803900 -0.66632200 0.05151800
C 25.19883800 0.32463000 -0.18933600
O 24.99600800 1.44763800 -0.66247200
N 26.42165400 -0.13716200 0.15324200
H 26.56670500 -1.06640800 0.54289800
C 27.65545600 0.62448200 -0.05325000
C 28.81446700 -0.38212700 0.17327100
O 28.60310300 -1.50808900 0.63465400
N 30.04349200 0.06820500 -0.16814900
H 30.20001600 1.00132300 -0.54320600
C 31.26344400 -0.71316400 0.03705400
C 32.44157400 0.27336600 -0.16856600
O 32.26212700 1.40482300 -0.61187200
N 33.66868400 -0.19911300 0.19002800
H -26.60421200 -0.90661100 1.22653800
H -22.98810700 0.68167900 -1.28443200
H -19.36790800 -0.80764100 1.29048100
H -15.75334000 0.77225300 -1.24077400
H -12.12950100 -0.75993000 1.31034600
H -8.51687500 0.85156700 -1.20716200
H -4.89194400 -0.75633300 1.29736300
H -1.27937000 0.91571800 -1.18143200
H 2.34474200 -0.79136800 1.25823000
H 5.95955000 0.96257400 -1.16015300
H 9.58116100 -0.85305000 1.20315800
H 13.20009900 0.98840500 -1.14240300
H 16.81725400 -0.92808700 1.14526600
H 20.44187400 0.99241900 -1.12468600
H 24.05146100 -1.00811100 1.09715100
H 27.68293100 0.98084200 -1.09392000
H 31.27879500 -1.08252700 1.07374900
N -31.49099500 -0.30080200 0.05306000
H -30.20624800 0.62982800 -1.31135300
H 33.80671200 -1.14561400 0.51439200
H 34.47582300 0.38548200 0.01785500
C -30.26665900 0.44487200 -0.22820500
C -32.72116900 0.11134000 -0.38451800
O -32.89504000 1.13565500 -1.04979600
H -31.37353300 -1.13010900 0.62835600
C 31.41056800 -1.90122300 -0.88259600
H 30.50759300 -2.55876200 -0.80273200
H 32.30522200 -2.50221200 -0.58965800
H 31.52514000 -1.58567600 -1.94775100
C 27.81377900 1.82135000 0.85187500
H 26.92224100 2.49201200 0.75473400
H 28.72118000 2.40383200 0.55936300
H 27.91365200 1.51819300 1.92204200
C 24.17135000 -1.87746900 -0.83679000

H 23.27123000 -2.53523700 -0.73133800
H 25.07105400 -2.46732500 -0.53549500
H 24.27598700 -1.59022300 -1.91089700
C 20.57885600 1.89429100 0.79319700
H 19.68623500 2.56097100 0.68016500
H 21.48412900 2.46805200 0.47772800
H 20.68495300 1.62465400 1.87170900
C 16.93598100 -1.86190600 -0.75854500
H 16.03664400 -2.51689900 -0.63141600
H 17.83623800 -2.44050500 -0.43759500
H 17.04059800 -1.61055600 -1.84161200
C 13.33921500 1.95830700 0.74193500
H 12.44502900 2.61895700 0.60792000
H 14.24248200 2.52226300 0.40380600
H 13.44885400 1.72723500 1.82901200
C 9.70193700 -1.86000200 -0.66295200
H 8.80368200 -2.51125200 -0.51129600
H 10.60289800 -2.42420000 -0.31912300
H 9.80708900 -1.65060500 -1.75485300
C 6.09573500 2.00783900 0.68372400
H 5.19981600 2.66025100 0.52351300
H 6.99747600 2.56017100 0.32307100
H 6.20610900 1.82084900 1.77917100
C 2.46886300 -1.87382200 -0.56494200
H 1.57220600 -2.52068600 -0.38725600
H 3.37103700 -2.42111000 -0.19774400
H 2.57425900 -1.70909000 -1.66444400
C -1.15129000 2.03340000 0.62007100
H -2.04888600 2.67599200 0.43197900
H -0.25035300 2.57401600 0.24017300
H -1.04320000 1.89015000 1.72231800
C -4.76353900 -1.90500900 -0.48450300
H -5.65809500 -2.54786600 -0.28321600
H -3.85963200 -2.43526100 -0.09713300
H -4.65831900 -1.78067500 -1.58932000
C -8.40086200 2.02695900 0.55800700
H -9.29994900 2.65965300 0.34510500
H -7.50006500 2.55845400 0.16514200
H -8.29755100 1.91965700 1.66479000
C -11.99518200 -1.95629300 -0.43932200
H -12.88734100 -2.59684700 -0.22077400
H -11.08932800 -2.47208000 -0.03727400
H -11.88999000 -1.86186000 -1.54709700
C -15.65130700 1.98477700 0.49982400
H -16.55152100 2.60920800 0.26810400
H -14.75007900 2.51126200 0.10128700
H -15.55443600 1.90127500 1.60923500
C -19.22674100 -2.02976500 -0.44052200
H -20.11643600 -2.67039000 -0.21234200
H -18.31886300 -2.53544700 -0.03036300
H -19.12166000 -1.95194300 -1.54959200
C -22.90176900 1.90676100 0.44777200
H -23.80228600 2.52616200 0.20401200
H -21.99904100 2.43310200 0.05258200
H -22.81374300 1.83188700 1.55851600
C -26.46008000 -2.12084000 -0.50894700
H -27.34634800 -2.76572700 -0.27960400
H -25.54833200 -2.62343500 -0.10388300

```
H -26.35970700 -2.03874500 -1.61811700
C -30.16348500 1.77933200 0.47131100
H -29.24907000 2.32082700 0.12727200
H -30.11126300 1.65955600 1.58026300
H -31.05732900 2.40657200 0.22316200
C -33.88271900 -0.77805500 -0.01333300
H -33.98950100 -0.84355300 1.09623500
H -33.74102000 -1.80605700 -0.42567700
H -34.81863400 -0.34304300 -0.44128500
```

α-helix-Ala$_{18}$

```
C 12.98677500 -0.51314500 -1.81705000
C 11.87201900 -1.02213700 -0.87032300
N 12.24586300 -1.38290400 0.38246700
C 11.24661600 -1.86670300 1.35635700
C 10.19196000 -0.78588800 1.72908600
N 10.62477100 0.49857400 1.75973500
C 9.67929400 1.61121500 1.98639900
C 8.65322600 1.74849500 0.82901400
N 9.15717500 1.64286800 -0.42411200
C 8.26755800 1.63258700 -1.60211700
C 7.23061900 0.47529600 -1.53677900
N 7.71431300 -0.73560200 -1.17650900
C 6.81634100 -1.89697400 -1.02272300
C 5.71765500 -1.65073400 0.04856300
N 6.12981100 -1.10265800 1.21221100
C 5.16522800 -0.76635000 2.27649700
C 4.09130000 0.24763100 1.79372500
N 4.54432100 1.29920600 1.07805300
C 3.61119800 2.29133800 0.51319400
C 2.59714000 1.64909500 -0.47370300
N 3.10737900 0.77081600 -1.36305300
C 2.23059600 0.04353500 -2.29891000
C 1.17221600 -0.82052700 -1.55858800
N 1.62484200 -1.54727500 -0.51491500
C 0.70094300 -2.34737400 0.30890300
C -0.39721600 -1.47304600 0.97654400
N 0.02419600 -0.32235900 1.54201100
C -0.93256900 0.62758600 2.13815000
C -1.97932300 1.13310500 1.10666800
N -1.49625500 1.49421500 -0.10088900
C -2.40088800 1.92284400 -1.18237000
C -3.42160200 0.81629500 -1.57091900
N -2.92262200 -0.43278600 -1.68405300
C -3.79846900 -1.58172000 -1.97459400
C -4.88639300 -1.78702800 -0.88363100
N -4.47048000 -1.68651400 0.39677200
C -5.42972400 -1.78317300 1.51140900
C -6.50421400 -0.65959300 1.46519100
N -6.05091700 0.57779200 1.16922200
C -6.98405900 1.70915800 1.03236000
C -8.02192400 1.48486100 -0.10171300
N -7.53931800 0.99160700 -1.26383800
C -8.45083900 0.72588300 -2.38980700
C -9.45558800 -0.42525300 -2.09839400
N -8.95609600 -1.49410100 -1.43572200
C -9.80741300 -2.65891900 -1.14728700
```

C -10.87918200 -2.40522500 -0.04835400
N -10.54795500 -1.49232800 0.90760700
C -11.46355900 -1.21027900 2.02096800
C -12.37758100 0.03264800 1.81394600
N -11.94815300 0.95777600 0.90599100
C -12.65979000 2.22951700 0.72356900
C -13.17024400 2.48162500 -0.72103500
N -12.85623700 1.54852200 -1.65688400
O 10.70924900 -1.09636900 -1.27416300
O 9.03972800 -1.12990900 2.00517800
O 7.45681600 1.93595700 1.06634400
O 6.04311400 0.68700100 -1.80170800
O 4.54214000 -1.95320100 -0.18655000
O 2.89919000 0.07127500 2.07299700
O 1.39829300 1.95084100 -0.41898100
O -0.00664300 -0.82103900 -1.93519800
O -1.57809400 -1.84386800 0.96963400
O -3.17738000 1.19084700 1.41192900
O -4.61036100 1.10385900 -1.76067500
O -6.05508800 -2.03241400 -1.20743500
O -7.69162400 -0.91670700 1.69568100
O -9.21347000 1.76475900 0.07453600
O -10.62720200 -0.34747300 -2.48511800
O -11.93915700 -3.02988900 -0.07359600
O -13.42064400 0.13005800 2.46259000
O -13.84250100 3.48637100 -0.96842600
N 14.02299800 0.27230900 -1.10772900
H 12.46240800 0.13648400 -2.53052900
H 13.21864000 -1.29831300 0.65026000
H 10.66263600 -2.66896900 0.88906900
H 11.52341700 0.72905700 1.34718500
H 9.07571300 1.37863100 2.87033200
H 10.15320600 1.49618900 -0.55297300
H 7.65972000 2.54561200 -1.60366900
H 8.71741000 -0.86647100 -1.06259100
H 6.25726700 -2.05397500 -1.95402800
H 7.12166700 -0.94864100 1.38594500
H 4.59154100 -1.66161800 2.54854300
H 5.54493400 1.42741800 0.93350000
H 2.98531200 2.70378300 1.31491100
H 4.11351000 0.61147700 -1.40556500
H 1.63716900 0.76126400 -2.87982700
H 2.62003000 -1.55795800 -0.29213400
H 0.14161200 -3.03928000 -0.33391500
H 1.02028000 -0.10419700 1.57785000
H -1.53100300 0.11651600 2.90340300
H -0.48996600 1.49601800 -0.27050500
H -3.02496300 2.75530600 -0.83219400
H -1.91956900 -0.59256100 -1.58772000
H -4.36866300 -1.38704800 -2.89210700
H -3.47966700 -1.56073700 0.60539000
H -6.00585900 -2.71289700 1.41956400
H -5.05036000 0.74975600 1.07132400
H -7.59155300 1.79656000 1.94219400
H -6.53548000 0.86146100 -1.38966300
H -9.08638200 1.60519800 -2.55374600
H -7.96025100 -1.54900300 -1.22436700
H -10.39843800 -2.89686700 -2.04064200

H -9.58406400 -1.17118700 0.97783900
H -12.16368600 -2.05109000 2.09171100
H -10.98348800 0.92013100 0.58204200
H -13.56059500 2.17005300 1.34698600
H -12.20973800 0.78346400 -1.49922800
H -13.13786500 1.74014100 -2.60824200
C 13.67740800 1.44381200 -0.47677000
O 12.49785500 1.74877600 -0.25895000
H 14.99283300 0.15184000 -1.37968200
C 14.80323100 2.33093800 -0.02451100
H 14.38249300 3.17789200 0.57233700
H 15.53248900 1.77109100 0.61009900
H 15.34122800 2.75124300 -0.90972500
C 13.57217300 -1.68466200 -2.56040300
H 12.74481200 -2.24624200 -3.06321400
H 14.10789000 -2.38641600 -1.87523200
H 14.28902900 -1.34037900 -3.34625300
C 11.87796300 -2.38896000 2.61885400
H 11.06840700 -2.73078400 3.31226600
H 12.54532500 -3.25875700 2.40086100
H 12.47721500 -1.60295800 3.14044200
C 10.38779800 2.92428700 2.18492700
H 9.62784500 3.73931600 2.27946000
H 11.00139400 2.90390800 3.11881700
H 11.06184600 3.16230700 1.32304200
C 9.03900000 1.53811100 -2.89173000
H 8.31923400 1.47882500 -3.74555300
H 9.68202200 2.44104800 -3.03488400
H 9.69257200 0.62899200 -2.91284300
C 7.57448900 -3.15110600 -0.67420100
H 6.84925200 -3.99044900 -0.53362500
H 8.28148600 -3.42723500 -1.49478400
H 8.16166100 -3.02534100 0.27092400
C 5.85114200 -0.21584500 3.49939500
H 5.08056900 0.04758200 4.26513900
H 6.54564400 -0.97419400 3.93814100
H 6.44340000 0.70276200 3.25580800
C 4.33856800 3.41042500 -0.18541500
H 3.59138900 4.11460800 -0.62732200
H 4.97861300 3.97603900 0.53565800
H 4.99364500 3.02059000 -1.00585200
C 3.01748600 -0.83600900 -3.23533600
H 2.30990900 -1.39585700 -3.89505100
H 3.69440400 -0.22151200 -3.87844600
H 3.64307600 -1.57410300 -2.67118900
C 1.42964400 -3.12381400 1.37460900
H 0.68641600 -3.68088000 1.99631100
H 2.13360500 -3.86018000 0.91462900
H 2.01889800 -2.44410300 2.04197500
C -0.23390500 1.81291500 2.75218900
H -0.99821900 2.52180300 3.15518100
H 0.42996100 1.48849200 3.59095000
H 0.39340100 2.35162600 1.99652600
C -1.64100900 2.34405700 -2.41339000
H -2.36726400 2.62294300 -3.21580000
H -0.99348200 3.22838200 -2.19415000
H -0.98811500 1.51596500 -2.79097100
C -3.01214300 -2.85778200 -2.12978600

H -3.71833100 -3.70459500 -2.31177900
H -2.30927500 -2.78536200 -2.99588400
H -2.41306800 -3.07987200 -1.20965600
C -4.73905000 -1.74512900 2.84993600
H -5.50760700 -1.78267900 3.66049900
H -4.05374000 -2.62037700 2.96595200
H -4.13624200 -0.80878500 2.96918400
C -6.25802400 3.00578600 0.78157000
H -7.00568000 3.82560000 0.64810500
H -5.59687800 3.26118800 1.64586200
H -5.62307300 2.94405900 -0.13912800
C -7.70250800 0.39987500 -3.65681700
H -8.43767900 0.18257500 -4.47023200
H -7.06434600 1.26150300 -3.97204800
H -7.04299800 -0.49400700 -3.52134200
C -8.99858900 -3.86679500 -0.74731800
H -9.69374500 -4.71410200 -0.52753700
H -8.30809500 -4.17119600 -1.57166100
H -8.38262200 -3.66154000 0.16345700
C -10.74864400 -1.04716800 3.33944900
H -11.48503400 -0.73349800 4.11986800
H -10.28339900 -2.01334800 3.65323000
H -9.93865000 -0.27858500 3.27603400
C -11.85571000 3.43667500 1.14318400
H -12.42465100 4.36417700 0.88598400
H -11.67356600 3.42160900 2.24510100
H -10.86485700 3.46502700 0.62422700

3_{10}-helix-Ala$_{18}$

C -18.56587600 -1.13441900 -1.55640900
H -18.69560100 -2.02055400 -0.93568000
C -17.42202700 -0.29559100 -1.00899400
O -16.30320300 -0.79997900 -0.94400300
H -18.33744200 -1.43676100 -2.57784400
N -17.67704700 0.95516200 -0.61941900
H -18.61954600 1.31141800 -0.69899400
C -16.64178100 1.81847500 -0.08687500
H -16.33275600 2.47518600 -0.90015400
C -15.45368600 1.00665000 0.40960000
O -14.34590800 1.14728700 -0.10143600
C -17.16398600 2.64431900 1.08306700
H -16.36623800 3.28227900 1.46281500
H -17.99570000 3.26351700 0.74868300
H -17.50377200 1.97743000 1.87616700
N -15.68955400 0.15401800 1.41037900
H -16.62353600 0.08539500 1.78597900
C -14.64159000 -0.67534300 1.97117800
H -14.31419800 -0.18797000 2.88979800
C -13.47291300 -0.81009800 1.00573500
O -12.35797600 -0.39522400 1.31468900
C -15.15670300 -2.07793100 2.27756900
H -14.35023000 -2.67790600 2.69717900
H -15.97461800 -2.01603400 2.99502400
H -15.51407200 -2.54219400 1.35839900
N -13.73037600 -1.39201600 -0.16774400
H -14.66876500 -1.71079000 -0.36112300
C -12.70277100 -1.57866400 -1.17215600

H -12.37337500 -2.61419500 -1.09393600
C -11.52970100 -0.63736400 -0.93688100
O -10.40953700 -1.08401600 -0.70081400
C -13.24568100 -1.30545600 -2.57053500
H -12.45280400 -1.45312700 -3.30389000
H -14.06762800 -1.98985300 -2.78085100
H -13.60497900 -0.27802400 -2.62659100
N -11.79045800 0.66997300 -1.00224700
H -12.73299300 0.97338900 -1.20016900
C -10.75830800 1.66718600 -0.79706500
H -10.44856400 2.00520400 -1.78620200
C -9.57020100 1.08026700 -0.04905900
O -8.45955100 1.04067800 -0.57506700
C -11.28676600 2.84333700 0.01717500
H -10.49179900 3.57595900 0.15529400
H -12.11940800 3.30757700 -0.51238200
H -11.62698300 2.48873600 0.99036100
N -9.80552100 0.62293700 1.18299900
H -10.74115600 0.68464800 1.55797600
C -8.75725500 0.04144300 1.99698400
H -8.43424900 0.81601900 2.69190700
C -7.58484400 -0.41240300 1.13816600
O -6.47234900 0.08726200 1.28865100
C -9.26898900 -1.17206900 2.76572100
H -8.46112500 -1.58853000 3.36792200
H -10.08978900 -0.87024400 3.41666900
H -9.62250600 -1.92572200 2.06200600
N -7.83772700 -1.36378200 0.23591900
H -8.77513400 -1.73362700 0.16303800
C -6.80684400 -1.88041000 -0.64002300
H -6.47386800 -2.82500500 -0.21053000
C -5.63678700 -0.91208600 -0.74125000
O -4.51571500 -1.24589200 -0.36386800
C -7.34679100 -2.10752900 -2.04876600
H -6.55076000 -2.49508600 -2.68482600
H -8.16462400 -2.82537500 -2.01255000
H -7.70885200 -1.16355400 -2.45546300
N -5.90078100 0.29227800 -1.25323400
H -6.84352900 0.50587300 -1.54463400
C -4.87290200 1.30339600 -1.40173900
H -4.56075700 1.28225500 -2.44606200
C -3.68415000 1.01386100 -0.49487600
O -2.57282600 0.80060900 -0.97303100
C -5.40672300 2.68641000 -1.04278500
H -4.61407700 3.42398200 -1.16280200
H -6.23873700 2.93592800 -1.69995000
H -5.74848800 2.68627000 -0.00739800
N -3.92253600 1.00714100 0.81861800
H -4.85962400 1.19067100 1.14790000
C -2.87537600 0.74540600 1.78527200
H -2.55645700 1.71236800 2.17244200
C -1.69890700 0.02778000 1.13764900
O -0.58779600 0.55311900 1.10791200
C -3.38532600 -0.13182000 2.92353000
H -2.57855500 -0.31217500 3.63350100
H -4.20840000 0.37260900 3.42987300
H -3.73565800 -1.08268800 2.52102200
N -1.94643400 -1.17620100 0.61643800

H -2.88168300 -1.55198300 0.67275100
C -0.91127400 -1.95923700 -0.02700100
H -0.57693000 -2.69669100 0.70269100
C 0.25592700 -1.07979200 -0.45255000
O 1.37709600 -1.25909600 0.01887000
C -1.44564200 -2.65850200 -1.27188500
H -0.64716300 -3.23830800 -1.73418900
H -2.26310300 -3.32378100 -0.99266700
H -1.80967500 -1.91321900 -1.97989200
N -0.00958200 -0.12550800 -1.34821000
H -0.95171100 -0.02934800 -1.69693900
C 1.01549400 0.77608700 -1.83315700
H 1.33119700 0.39815400 -2.80548800
C 2.20229900 0.82037600 -0.88043100
O 3.31592400 0.45882200 -1.25374700
C 0.47685600 2.19559300 -1.97264600
H 1.26802300 2.85016000 -2.33795000
H -0.35385900 2.20113100 -2.67787400
H 0.13153200 2.55022900 -1.00119300
N 1.96004400 1.26543200 0.35457500
H 1.02185700 1.54696300 0.59993100
C 3.00564600 1.35420500 1.35426900
H 3.32089200 2.39785700 1.38609900
C 4.18611000 0.46337900 0.99475500
O 5.29552400 0.95004300 0.78878700
C 2.49463600 0.92122200 2.72427100
H 3.29920200 0.99857100 3.45397100
H 1.66801300 1.56616500 3.02491500
H 2.14915100 -0.11106100 2.67294100
N 3.94363100 -0.84764200 0.92044200
H 3.00868300 -1.18417300 1.10051000
C 4.98317000 -1.80010200 0.58641100
H 5.31699600 -2.24037100 1.52628800
C 6.14937800 -1.11636200 -0.11376300
O 7.26877500 -1.11840200 0.39360500
C 4.45428400 -2.88658900 -0.34279200
H 5.25568700 -3.58717100 -0.57584000
H 3.63864400 -3.41862400 0.14688500
H 4.08999600 -2.43160200 -1.26397500
N 5.88277500 -0.52931400 -1.28257100
H 4.94168100 -0.56302600 -1.64483300
C 6.90716500 0.15484700 -2.04646700
H 7.22589900 -0.53358900 -2.82935500
C 8.09052200 0.52827600 -1.16459000
O 9.20618400 0.06411400 -1.38960700
C 6.36451300 1.43728700 -2.66642900
H 7.15510400 1.92953300 -3.23358800
H 5.53542400 1.19663500 -3.33228400
H 6.01603900 2.10353300 -1.87681800
N 7.84406800 1.36988000 -0.15791800
H 6.90406000 1.71516700 -0.02703000
C 8.88621900 1.80150900 0.75102900
H 9.19757000 2.79249300 0.42218200
C 10.06987500 0.84537000 0.72202900
O 11.17803200 1.23542600 0.36342300
C 8.37123100 1.86303200 2.18578500
H 9.17443700 2.19073200 2.84550700
H 7.54251100 2.56903100 2.24445200

```
H 8.02979100 0.87497200 2.49202400
N 9.83161400 -0.41237000 1.10199600
H 8.89762300 -0.67033100 1.38588000
C 10.87412500 -1.41792000 1.11866500
H 11.20693100 -1.50759400 2.15315800
C 12.04105000 -1.01181000 0.22821900
O 13.15961700 -0.83577800 0.70695700
C 10.35191000 -2.75977600 0.61895000
H 11.15606000 -3.49500400 0.64206300
H 9.53581500 -3.09408200 1.25990400
H 9.98906800 -2.65087400 -0.40329000
N 11.77647600 -0.86449000 -1.07192100
H 10.83568000 -1.02376400 -1.40282800
C 12.80126100 -0.48044200 -2.02228600
H 13.12452100 -1.39458600 -2.51978900
C 13.98069600 0.17728100 -1.32077800
O 15.09818700 -0.33022800 -1.37061900
C 12.25674500 0.50862000 -3.04632200
H 13.04717900 0.77818700 -3.74717800
H 11.43046500 0.05109700 -3.59087700
H 11.90325500 1.40388100 -2.53469200
N 13.72874700 1.31325100 -0.66544900
H 12.78693700 1.67962200 -0.66306500
C 14.76664200 2.03492700 0.04136300
H 15.07586400 2.85422000 -0.60747400
C 15.95309700 1.13159600 0.34589600
O 17.06183700 1.37944700 -0.12340100
C 14.24759600 2.58458000 1.36640000
H 15.04770200 3.12192500 1.87521300
H 13.41680600 3.26507600 1.17730100
H 13.90786600 1.76088000 1.99290400
N 15.71754200 0.08129600 1.13472700
H 14.78321800 -0.06765400 1.48822800
C 16.76379700 -0.85426500 1.49717500
H 17.09364500 -0.58115800 2.50039200
C 17.93069100 -0.77464500 0.52410600
O 19.04793500 -0.44046600 0.91474200
C 16.24619800 -2.28839300 1.48851400
H 17.05270000 -2.96749500 1.76532400
H 15.42871200 -2.38414200 2.20384800
H 15.88608700 -2.53884100 0.49046600
N 17.67075500 -1.08389000 -0.74788900
H 16.73132700 -1.35071900 -1.00557800
H 18.37876000 -1.05849500 -1.45364000
H -19.46659700 -0.55689700 -1.54801500
```


(1) Kundu, S.; Saha, A. Electrostatically Embedded Grid-Adapted Many-Body Analysis (EE-GAMA): A Charge Embedded Fragment-Based Quantum Chemistry Method for Accurate Modelling of Neutral and Charged Molecular Clusters. *Comput. Theor. Chem.* **2025**, *1254* (August), 115534. https://doi.org/10.1016/j.comptc.2025.115534.
