# Supplementary material for: GAMA: A Robust and Automated Fragment-Based Quantum Chemistry Method for Biomolecular Systems
Source: J Phys Chem Lett. 2026 Mar 18;17(13):3736–41. doi: 10.1021/acs.jpclett.5c03778 (PMC13051465; doi:10.1021/acs.jpclett.5c03778)
Supplement: Supplementary file 2 [file jz5c03778_si_002.pdf]

jz-2025-03778a.R1

Name: Peer Review Information for "GAMA: A Robust and Automated Fragment-Based Quantum Chemistry Method for Biomolecular Systems"

First Round of Reviewer Comments

Reviewer: 1

Comments to the Author

This letter provides benchmark calculations on biological systems for the GAMA2 fragmentation method, which was introduced in previous works (Refs. 7 and 8). Whereas previous tests of this method did not sever covalent bonds, these systems require breaking and capping C-C bonds in the peptide backbone. Additionally, this letter explores the use of density functional theory (DFT)-based methods for the full-system correction.

This work highlights potentially useful developments in affordable and systematically improvable fragmentation methods. The focus on biological systems may be of interest to those outside of the fragmentation community.

This letter will require some major revisions as outlined below. Once those completed, I would recommend this work for publication in JPC Letters.

Comments:

- This work indicates significant accuracy improvements from the use of DFT-based methods instead of Hartree-Fock (HF). As the authors articulated in their previous work (Ref. 7), without a counterpoise correction, this may simply be error cancellation. For example, correlation is accounted for both in MP2 and DFT; perhaps there is some double counting occurring? Additionally, DFT-based fragmentation methods suffer from

fragmentation-specific issues with self-interaction error which can also be mitigated using a counterpoise correction (DOI:10.26434/chemrxiv-2025-r6t19, DOI:10.1039/D4SC05955G).

- When discussing computational costs, the wall time is reported but the CPU time is not. Including the CPU time would make it easier to compare GAMA to other fragmentation methods. As an aside, for practical large-scale simulations resolution of identify (RI)-MP2 is frequently used drastically improving timings (DOI:10.1063/1.4927325, DOI:arXiv:2410.21888v1)

- As pointed out in Ref. 7, GAMA2 is more performant than MBE(3) due to the omission of most 3-body (in the MBE sense) terms. It may be a valuable comparison to MBE-based methods to show how many of the GAMA fragments contain 1, 2, or 3 amino acid residues.

- Two-body GAMA terms are screened using a distance cutoff. Would the authors clarify how this cutoff is defined in GAMA? For example, is it the distance between the cell edges or is it the closest approach between the "groups of atoms".

- The authors state that GAMA2 "demonstrates good performance" but this is not elaborated on. Would the other please briefly discuss how they anticipate this algorithm being used and what level of accuracy these applications may require?

- The GAMA algorithm partitions the chemical system onto grid cells with "groups of atoms" potentially mapping onto multiple cells. This may lead to algorithmic edge cases such as when a segment transitions from spanning two cells to only one. Would the authors be able to comment on or quantify the magnitude of these effects?

Minor Issues:

- On pg. 5, it is noted that MBE and FMO3 require "tens to hundreds of CPU hours...". Later in that paragraph GAMA2 is described as requiring only a ~1.17 hour wall. Although the

latter number was a direct comparison to the full MP2 cost (192 hours), it would make it easier to interpret if all three numbers had the same unit.

- The pg. 5 includes a of survey of fragmentation methods applied peptides of different lengths. For this section, reporting errors in kcal/mol/peptide may be a more size intensive unit.

- Acronyms are frequently undefined. Some examples are (e.g. MFCC, FMO, MBE)

- Units are missing from Tables S1-4

Reviewer: 2

Comments to the Author

This manuscript provides an overview of the Grid-Adapted Many-Body Analysis (GAMA) fragmentation scheme for carrying out calculations on large molecules. The work highlights the model's accuracy, presents a simple and significant improvement in accuracy by using a more accurate low-level model chemistry, and presents a demonstration of computational speed-up provided by GAMA2.

Overall, the manuscript is well written and presents a meaningful and impactful report that will be of interest to JPC Letters readers.

I suggest the authors consider the following comments when revising the manuscript.

1. Overall, the manuscript would benefit from describing the key impact of the paper earlier in the narrative. The background and motivation provided at the start is important, but it

would be helpful to more prominently present the goals and impact of the new work presented in the manuscript.

2. Is the authors' GAMA software freely available? If so, I suggest saying so in the manuscript and pointing interested readers to the location.

3. In the comparison of computational timings, the authors use 40 nodes for their GAMA calculations but only use 1 node in the benchmark calculation. The results described in the paper should present these data on equal footing. One option would be to show the cost of an MP2 calculation using 40 nodes, though that may lead to a conversation focused on the scaling of the employed MP2 implementation. As an alternative, one could include a comment about the best-case scenario of linear scaling across 40 nodes. In such a case, the MP2 calculation time could be projected to be ~4 hours. That would lead to a different speed-up afforded by GAMA than the 164-fold speed-up reported in the manuscript.

4. It seems that MFCC and FBQC may be used in the manuscript without providing their definitions, which should be addressed.

5. Figure 3 shows data for three different systems, but the captions refers to two systems.

Reviewer: 3

Comments to the Author

Review of the manuscript "GAMA: A Robust and Automated Fragment-Based Quantum

Chemistry Method for Biomolecular Systems" by Kundu and Saha.

The paper presents accuracy and computational benchmarks for GAMA (Grid-Adapted Many-Body Analysis) on biomolecular systems. GAMA performs full-system low-level calculations (HF or DFT) and high-level (MP2) calculations on H-capped fragments, which are automatically prepared based on the selected grid size. The benchmark systems include medium-size polypeptides (including 1YlP, beta-strand Ala18, alpha-helix Ala18, Gly12) with different H-bonding patterns and polarization. The accuracy tests demonstrate

that GAMA can reproduce full-system MP2 results with errors up to 13 kcal/mol when HF is used as the full-system low-level method, and 4-5 kcal/mol when DFT functionals are used as the low-level method. The authors indirectly compare GAMA's accuracy with that of other fragmentation methods (MFCC, FMO, SMF, MBE) and claim that GAMA outperforms them. No direct comparisons are shown. Overall, while the development of an accurate and universal fragmentation model is undoubtedly of great demand, the present manuscript does not present sufficient evidence that GAMA will fill this gap. The specific comments are below.

1. GAMA is limited to mid-system sizes because it is based on full-system calculations with HF or DFT, whereas the discussed fragmentation methods do not have this limitation and have been used to investigate much larger, physically and biologically relevant systems. It would be fairer to compare the performance and accuracy of GAMA with other methods based on the full-system HF method, such as SAPT versions (FSAPT, XSAPT) or ONIOM models.

2. It is unclear how GAMA could be applied to any properties rather than total system energies (interaction energies, gradients?). What are the examples of biological problems that GAMA could solve?

3. The claim of GAMA being "a practical and transformative tool for performing correlated quantum calculations on biomolecular systems that were previously intractable" is clearly an overstatement. As mentioned above, other fragmentation methods have been applied to significantly larger systems than will ever be possible with GAMA. Another question is whether MP2 provides higher accuracy than the new dispersion-corrected functions. Based on benchmarks on non-covalent databases, this is no longer the case, as MP2 overestimates dispersion interactions. Thus, what is the advantage of performing MP2 on top of DFT instead of using a good DFT as the only method?

4. The exact (rather than implicit) benchmarks of GAMA against other available solutions in the field are necessary for any realistic claims of superior accuracy.

Author's Response to Peer Review Comments:

Response to the reviewers' comments

We sincerely thank all the reviewers for their careful evaluation of our manuscript and for their insightful and constructive comments, which have significantly helped to improve the quality of our work. We have addressed all the comments from all three reviewers and incorporated the necessary revisions into the manuscript.

Reviewer: 1

Recommendation: This paper is probably publishable, but major revision is needed; I do not need to see future revisions.

This letter provides benchmark calculations on biological systems for the GAMA2 fragmentation method, which was introduced in previous works (Refs. 7 and 8). Whereas previous tests of this method did not sever covalent bonds, these systems require breaking and capping C-C bonds in the peptide backbone. Additionally, this letter explores the use of density functional theory (DFT)-based methods for the full-system correction. This work highlights potentially useful developments in affordable and systematically improvable fragmentation methods. The focus on biological systems may be of interest to those outside of the fragmentation community. This letter will require some major revisions as outlined below. Once those completed, I would recommend this work for publication in JPC Letters.

We sincerely thank the reviewer for the thorough evaluation of our manuscript, for recognizing the novelty of our work, and for providing constructive and insightful comments. We have addressed all of the reviewer's specific comments in detail below.

Addressing the specific comments of reviewer 1

1. ***This work indicates significant accuracy improvements from the use of DFT-based methods instead of Hartree-Fock (HF). As the authors articulated in their previous work (Ref. 7), without a counterpoise correction, this may simply be error cancellation. For example, correlation is accounted for both in MP2 and DFT; perhaps there is some double counting occurring? Additionally, DFT-based fragmentation methods suffer from fragmentation-specific issues with self-interaction error which can also be mitigated using a counterpoise correction (DOI:10.26434/chemrxiv-2025-r6t19, DOI:10.1039/D4SC05955G).***

Response to the comment 1: We thank the reviewer for raising this important point regarding the use of DFT-based corrections and the potential role of error cancellation. In the present GAMA2 framework, the improved performance obtained with DFT-based low-level corrections does not arise from double counting of correlation effects. The total GAMA2 energy is constructed as- $E_{\text{GAMA2}} = E_{\text{frag}}^{\text{MP2}} + (E_{\text{full}}^{\text{DFT}} - E_{\text{frag}}^{\text{DFT}})$,
such that the DFT contribution enters only through a differential correction term. Because the fragment-level DFT energy is explicitly subtracted from the full-system DFT energy, the correlation component inherent to DFT does not accumulate on top of the MP2 correlation. Instead, the DFT term serves to recover long-range and inter-fragment effects that are missing from the truncated MP2 many-body expansion, while the chemically local correlation is described at the MP2 level. As a result, there is no formal double counting of correlation energy in the GAMA2 formulation.

Regarding basis-set superposition error and error cancellation, our previous work[1] explicitly examined the impact of counterpoise corrections in noncovalent water clusters, where BSSE is known to be most pronounced. There, we showed that the overlapping-fragment construction inherent to GAMA significantly reduces BSSE sensitivity, even in strongly hydrogen-bonded systems. In the present work, the systems considered are covalently bonded peptides, for which BSSE effects are generally much less severe than in noncovalent clusters, due to the intrinsic sharing of basis functions across covalent bonds[2–5]. Consequently, the role of BSSE—and the need for explicit counterpoise correction—is further diminished in this context. The consistent and systematic improvement observed when replacing HF with DFT in the low-level correction therefore reflects a genuine improvement in the description of polarization and medium-range electronic effects rather than fortuitous error cancellation. Nevertheless, although BSSE effects are expected to be less dominant in covalently bonded systems, we plan to explicitly incorporate BSSE corrections in a separate future study to systematically assess their potential impact in covalently bonded peptide systems.

With respect to self-interaction error in DFT-based fragmentation approaches, we note that its impact is mitigated in GAMA2 by the differential nature of the correction term. Since both the full-system and fragment-level DFT energies are evaluated using the same functional, much of the self-interaction error cancels when forming the energy difference. Moreover, the chemically most sensitive regions are treated at the MP2 level, further limiting the influence of DFT self-interaction on the final energy. Overall, the improved accuracy observed for GAMA2 therefore does not stem from uncontrolled error

cancellation, but from a balanced combination of MP2-level treatment of local correlation and a physically motivated DFT-based correction that efficiently captures longer-range interactions more accurately than HF , while maintaining systematic error control.

2. ***When discussing computational costs, the wall time is reported but the CPU time is not. Including the CPU time would make it easier to compare GAMA to other fragmentation methods. As an aside, for practical large-scale simulations resolution of identity (RI)-MP2 is frequently used drastically improving timings (DOI:10.1063/1.4927325, DOI:arXiv:2410.21888v1)***

Response to comment 2: We sincerely thank the reviewer for this important comment. In response, in addition to the wall-clock time, we have now also reported the corresponding CPU times for both the GAMA2 calculations and the full MP2 calculations for both peptide systems. A detailed discussion of the CPU-time analysis has been added on **Page 8 (2nd paragraph)** of the revised manuscript, with the relevant changes highlighted in yellow for clarity. The CPU-time data are additionally summarized in **Figure S2** and **Tables S7** and **S8** in the revised supporting information.

In addition to the wall-clock time, we have also reported the corresponding CPU times for both the GAMA2 and full MP2 calculations. As shown in **Figure S2** and **Tables S7** and **S8**, a substantial reduction in computational cost is observed for GAMA2 relative to full MP2 when assessed in terms of CPU time. For example, the full MP2 calculation for the Alpha-helix-Ala_{18} system requires approximately **1178 CPU hours**, whereas the corresponding GAMA2 calculation requires only **~77 CPU hours**, corresponding to an approximately 15-fold reduction in CPU time. One important point to note is that, as shown in **Table S8**, the total CPU time for GAMA HF calculations is larger than that of the corresponding full HF calculations for both Gly_{12} and Alpha-helix-Ala_{18}. However, in terms of wall-clock time, GAMA HF consistently exhibits a lower computational cost than the full HF calculations. This behaviour is fully consistent with prior literature[6]. For example, Herbert and co-workers (Ref. 6) reported that fragment-based HF calculations can require substantially larger total CPU time than full HF calculations, while still yielding reduced wall-clock times due to efficient parallel execution (see Fig. 9 of Ref. 6). Thus, it is possible for fragment-based HF calculations to exhibit higher total CPU time but lower wall-clock time compared to full HF calculations. In contrast, GAMA MP2 calculations

show a substantial reduction in both CPU time and wall-clock time relative to full MP2 calculations. This behaviour arises from the steep $O(N^5)$ scaling of supersystem MP2 calculations, which is dominated by electron-correlation contributions. Within GAMA, the single, computationally demanding full-system MP2 calculation is replaced by many significantly smaller fragment MP2 calculations involving far fewer basis functions. As a result, the cumulative computational cost is dramatically reduced. Furthermore, because the fragment MP2 calculations are independent, they can be efficiently parallelized, leading to pronounced reductions in both CPU and wall-clock times.

Table S7. Computational **CPU** times (in hours) for a medium-sized peptide and a larger peptide obtained from full MP2 calculations and from the GAMA2 approach. All reported timings correspond to **CPU** time. Low-level corrections in GAMA2 were carried out using HF/6-311G(d,p). Here GAMA2 computational time is the sum of Full HF time, GAMA HF time and GAMA MP2 time. All GAMA calculations (both GAMA HF and GAMA MP2) were performed using box size 2Å and cutoff radius 5Å.

**Peptide system**	**Full MP2**	**GAMA2**
Gly_{12}	61.74	51.68
Alph-helix-Ala_{18}	1178.00	77.49

Table S8. Computational **CPU** times (in hours) for a medium sized peptide and a larger peptide for Full HF calculations, GAMA HF calculations and GAMA MP2 calculations. All calculations were performed using 6-311G(d,p) basis set.

**Peptide system**	**Full HF**	**GAMA HF**	**GAMA MP2**
Gly_{12}	0.72	14.62	36.34
Alph-helix-Ala_{18}	3.49	16.00	58.00

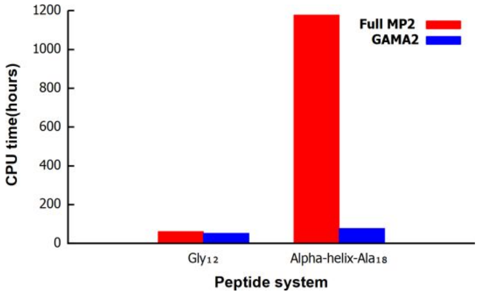

Figure S2. CPU times (in hours) for a medium-sized peptide (Gly_{12}) and a larger peptide (Alpha-helix-Ala_{18}) from full MP2 calculations versus the GAMA2 workflow. For GAMA2, the low-level correction uses HF/6-311G(d,p), and the total time includes the full-system HF step, the GAMA–HF correction, and the GAMA–MP2 fragment calculations. All GAMA calculations (both GAMA HF and GAMA MP2) were performed using box size 2Å and cutoff radius 5Å.

3. ***As pointed out in Ref. 7, GAMA2 is more performant than MBE(3) due to the omission of most 3-body (in the MBE sense) terms. It may be a valuable comparison to MBE-based methods to show how many of the GAMA fragments contain 1, 2, or 3 amino acid residues.***

   Response to comment 3: We sincerely thank the reviewer for this important comment. In the revised manuscript, we have addressed this point by analysing the distribution of GAMA fragment sizes for two peptides of different lengths, Gly_{12} (medium-sized peptide) and 3_{10}-helix-Ala_{18}. Specifically, we quantified the total number of GAMA fragments containing one, two, three, and four amino acid residues for both of the peptide systems. The corresponding fragment count distributions are reported in **Table S9** for the Gly_{12} peptide and **Table S10** for the 3_{10}-helix-Ala_{18} peptide. The associated histogram plots are provided in **Figure S3** (Gly_{12}) and **Figure S4** (3_{10}-helix-Ala_{18}).

   Table S9. Distribution of GAMA fragment counts by residue size for the Gly_{12} peptide obtained using a box size of 2 Å and a cutoff radius of 5 Å (total fragments = 94). The largest fragment contains four amino acid residues, while the smallest fragment contains a single residue.

**Fragment size**	**Number of fragments**
1 amino acid residues	15
2 amino acid residues	21
3 amino acid residues	36
4 amino acid residues	22

Table S10. Distribution of GAMA fragment counts by residue size for the 3_{10}-helix-Ala_{18} peptide obtained using a box size of 2 Å and a cutoff radius of 5 Å (total fragments = 104). The largest fragment contains four amino acid residues, while the smallest fragment contains a single residue.

**Fragment size**	**Number of fragments**
1 amino acid residues	18
2 amino acid residues	18
3 amino acid residues	37
4 amino acid residues	31

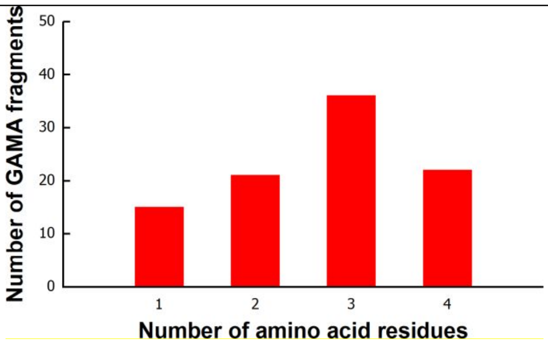

Figure S3. Histogram plot of the distribution of GAMA fragment counts by residue size for the Gly_{12} peptide obtained using a box size of 2 Å and a cutoff radius of 5 Å (total fragments = 94). The largest fragment contains four amino acid residues, while the smallest fragment contains a single residue. **Table S9** contains corresponding numbers.

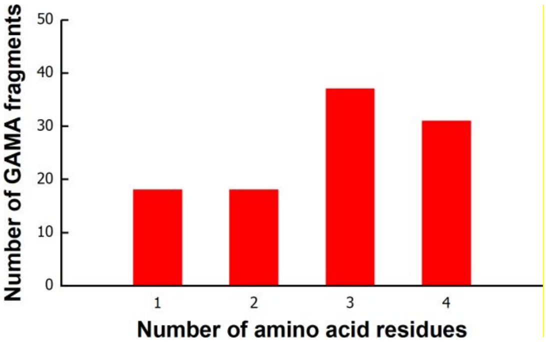

Figure S4. Histogram plot of the distribution of GAMA fragment counts by residue size for the Gly_{12} peptide obtained using a box size of 2 Å and a cutoff radius of 5 Å (total fragments = 94). The largest fragment contains four amino acid residues, while the smallest fragment contains a single residue. **Table S10** contains corresponding numbers.

4. ***Two-body GAMA terms are screened using a distance cutoff. Would the authors clarify how this cutoff is defined in GAMA? For example, is it the distance between the cell edges or is it the closest approach between the "groups of atoms".***

Response to comment 4: We thank the reviewer for this very important and insightful question. In the GAMA framework, the cutoff distance used in the two-body expansion is defined as the closest approach or the minimum interatomic distance between two “groups of atoms” belonging to different grid cells.

5. ***The authors state that GAMA2 "demonstrates good performance" but this is not elaborated on. Would the other please briefly discuss how they anticipate this algorithm being used and what level of accuracy these applications may require?***

Response to comment 5: We thank the reviewer for this insightful comment. In the revised manuscript, we have elaborated on the intended use and accuracy of GAMA2 algorithm on **page 10 (2nd paragraph)** of the revised manuscript, which is highlighted in yellow.

Specifically, GAMA2 is designed for applications where relative energetics, conformational preferences, and qualitative to semi-quantitative energetic trends are of primary interest, rather than sub–kcal mol^{-1} thermochemical accuracy. For flexible and moderately sized biomolecular systems, such as peptides and protein fragments, energy differences on the order of 1–4 kcal mol^{-1} are typically sufficient for reliable conformational ranking and structural analysis[7–10]. For more compact and highly cooperative systems, errors of ~2–5 kcal mol^{-1} still enable meaningful assessment of relative stability and energetic trends, particularly when full MP2 or higher-level calculations are computationally prohibitive[7–10]. In this context, GAMA2 provides a favourable balance between accuracy and computational efficiency, enabling correlated quantum-mechanical treatments of systems that are otherwise inaccessible with conventional supersystem methods. This combination of automation, accuracy, and computational efficiency makes GAMA2 a practical and systematically improvable framework for correlated wavefunction calculations on medium-sized biomolecular systems. More advanced electrostatically embedded variants, in which the full-system low-level correction is replaced by point-charge embedding, substantially reduce the computational cost and provide a pathway for extension of GAMA to larger biomolecular systems, which will be explored in future work.

6. ***The GAMA algorithm partitions the chemical system onto grid cells with "groups of atoms" potentially mapping onto multiple cells. This may lead to algorithmic edge cases***

such as when a segment transitions from spanning two cells to only one. Would the authors be able to comment on or quantify the magnitude of these effects?

Response to comment 6: We thank the reviewer for raising this important point regarding potential edge cases arising from the grid-based partitioning in the GAMA framework. We clarify below how such situations are treated in the present work.

In the current implementation of GAMA, overlap arises only incidentally due to the geometric extent of atomic groups relative to the underlying grid. Specifically, when a "group of atoms" spans more than one grid cell, it is naturally associated with multiple cells and therefore contributes to multiple fragments, defined as overlapping fragments. Conversely, when a group is fully contained within a single grid cell, it is treated solely as a primary fragment. Importantly, in the present work, intentional overlapping through the deliberate merging of adjacent grid cells is not imposed. Such intentional overlap will be explored in the future developments of the method to further explore accuracy and flexibility. Even in the presence of incidental overlap, the energetic contributions are treated in a rigorous and well-defined manner using the many-overlapping body expansion (MOBE) formalism. This framework[1,11] ensures that overlapping contributions are systematically accumulated while avoiding double counting, thereby enabling an accurate reconstruction of the total energy from fragment calculations. As a result, potential edge cases—such as when a group transitions from spanning two grid cells to residing within a single cell—do not introduce discontinuities or instabilities in the computed energies. Furthermore, the smooth convergence of total energies with respect to grid size, as demonstrated in the manuscript, indicates that such grid-induced effects are quantitatively minor in practice. These results support the robustness of the current GAMA implementation while also motivating future work in which intentional overlapping fragments will be explicitly introduced to further improve accuracy and transferability.

Minor comments

7. ***On pg. 5, it is noted that MBE and FMO3 require "hundreds to thousands of CPU hours...". Later in that paragraph GAMA2 is described as requiring only a ~1.17 hour wall. Although the latter number was a direct comparison to the full MP2 cost (192 hours), it would make it easier to interpret if all three numbers had the same unit.***

   Response to comment 7: We sincerely thank the reviewer for this helpful suggestion. We agree that presenting all computational timings using a consistent unit improves clarity and

facilitates a direct comparison. Since the computational costs for MBE and FMO3 in previous published references were reported in terms of CPU time, we have revised the corresponding paragraph to report the computational costs of both GAMA2 and full MP2 calculations in CPU hours. The relevant paragraph on **page 6**, now in the revised manuscript has been modified accordingly, and the changes have been highlighted in yellow in the revised manuscript. For convenience, we also provide the updated section below, with the modifications specifically highlighted.

These studies additionally highlight the considerable computational cost of several FBQC approaches[8–10]—particularly higher-order MBE, FMO3, and large-cutoff Systematic Molecular Fragmentation (SMF) approaches—which can require hundreds to thousands of CPU hours for 15–20 residue peptides. Importantly, because these published benchmarks employ varying fragmentation protocols and reference levels of theory, they do not allow for a direct side-by-side comparison with GAMA. Consequently, we do not claim that GAMA offers superior accuracy relative to these established benchmarks. Instead, these literature results serve to contextualize the broader challenges within the field and provide a frame of reference for our method's performance. Within this context, GAMA2 achieves total energy deviations of **0.01–4 kcal/mol** for flexible peptides (HF low-level) and **2–5 kcal/mol** for highly cooperative Alpha-helical-Ala_{18} and 3_{10}-helical-Ala_{18} systems (DFT low-level). Beyond accuracy, GAMA2 significantly optimizes efficiency: a full MP2 calculation for Alpha-helix-Ala_{18} peptide system requires ~**1,178 CPU hours**, whereas GAMA2 completes the task in just **~77 CPU hours** (**Figure S2**, **Table S7**) (*vide infra*). These results position GAMA2 as a systematically improvable fragmentation scheme that complements existing Fragment-Based Quantum Chemistry (FBQC) methods for covalently connected biomolecules.

8. ***The pg. 5 includes a survey of fragmentation methods applied peptides of different lengths. For this section, reporting errors in kcal/mol/peptide may be a more size intensive unit.***

   Response to comment 8: We thank the reviewer for this helpful suggestion. To provide a size-independent comparison across peptides of different lengths, we have reported, in addition to the total absolute errors, the corresponding errors in kcal/mol per peptide on **page 5**. The revised text highlights these changes in yellow. For convenience, we also provide the updated section below, with the modifications specifically highlighted.

Existing FBQC methods[7,8,10,12,13] have laid the strong foundation of this field in the last three decades providing an opportunity to make these methods more powerful. Towards that direction, we are focusing on existing challenges. Published benchmarks in the literature provide useful context regarding the challenges faced by FBQC methods for covalently bonded peptide systems. For example, Vornweg and co-workers[7] applied conventional MFCC and MFCC-MBE(2) schemes to three Ala_{10} peptide isomers (Alpha-helix, Beta-strand, and 3_{10}-helix) and reported deviations from supersystem MP2 energies: MFCC errors of ~ **60–80 kcal/mol** (~ **6–8 kcal/mol per peptide**) and MFCC-MBE(2) errors of ~ **6–8 kcal/mol** (~ **0.6–0.8 kcal/mol per peptide**) for the Alpha-helix and 3_{10}-helix isomers. Similar trends have been observed for Fragment Molecular Orbital (FMO) methods[14], which improve upon MFCC for polar or charged peptides but still show deviations of ~ **10–16 kcal/mol** (~ **0.5–2 kcal/mol per peptide**) for FMO2 and ~ **5–8 kcal/mol** (~ **0.2–0.8 kcal/mol per peptide**) for FMO3 in Alpha-helical polyalanine chains of 10–20 residues. SMF and MBE-with-capping approaches also show deviations of ~ **8–20 kcal/mol** (~ **0.8–2 kcal/mol per peptide**) for compact or Alpha-helical structures, reflecting incomplete recovery of higher-order polarization, charge-transfer, and cooperative hydrogen-bonding effects.

9. *Acronyms are frequently undefined. Some examples are (e.g. MFCC, FMO, MBE)*

   Response to comment 9 : We sincerely thank the reviewer for this important comment. We have fixed this in our revised manuscript, where we defined all the acronyms in the revised manuscript.

10. *Units are missing from Tables S1-4*

    Response to comment 10: We sincerely thank the reviewer for this comment. We have fixed this in the revised Supporting Information (SI) file.

Reviewer: 2

Recommendation: This paper may be publishable, but major revision is needed; I would like to be invited to review any future revision.

Comments: This manuscript provides an overview of the Grid-Adapted Many-Body Analysis (GAMA) fragmentation scheme for carrying out calculations on large molecules. The work highlights the model's accuracy, presents a simple and significant improvement in accuracy by using a more accurate low-level model chemistry, and presents a demonstration of

computational speed-up provided by GAMA2. Overall, the manuscript is well written and presents a meaningful and impactful report that will be of interest to JPC Letters readers. I suggest the authors consider the following comments when revising the manuscript.

Response: We sincerely thank the reviewer for the thorough evaluation of our manuscript and for the constructive feedback, which has significantly improved the quality of the work. We have addressed all the reviewer comments in a point-by-point manner, as detailed below.

Addressing the specific comments of reviewer 2

1. ***Overall, the manuscript would benefit from describing the key impact of the paper earlier in the narrative. The background and motivation provided at the start is important, but it would be helpful to more prominently present the goals and impact of the new work presented in the manuscript.***

   Response to comment 1: We sincerely thank the reviewer for this important comment. In response, we have now more prominently presented the major goal and impact of the work at the beginning of the manuscript (**page 2, 2nd paragraph**), and the relevant text has been highlighted in yellow.

   The major goal of this work is to develop a fully automated and systematically improvable fragment-based quantum chemical framework applicable to covalently bonded biomolecular systems, where fragmentation across covalent bonds poses a fundamental challenge. Building on our earlier Grid-Adapted Many-Body Analysis (GAMA) framework developed for noncovalently bound water clusters, we extend the GAMA protocol to covalently bonded peptide systems. The approach combines a grid-based fragmentation scheme, a many-body expansion truncated at the two-body level, and a low-level correction. The major impact of this work is the demonstration that GAMA2 (GAMA with two different layers) provides a controllable and efficient FBQC approach whose performance can be systematically tuned through the choice of low-level theory, grid box size, and interaction cutoff, enabling accurate MP2-level energies at substantially reduced computational cost and significantly advancing fragment-based treatments of large biomolecular systems.

2. ***Is the authors' GAMA software freely available? If so, I suggest saying so in the manuscript and pointing interested readers to the location.***

Response to comment 2: We sincerely thank the reviewer for this suggestion. We have made the full GAMA code for covalently bonded systems available on GitHub, along with all input files for the peptide systems used in this study. The repository can be accessed at the following link: [https://github.com/SahaLabGitHub/GAMA-peptide]. Details can be found at the "Data Availability Statement" of the revised manuscript.

3. ***In the comparison of computational timings, the authors use 40 nodes for their GAMA calculations but only use 1 node in the benchmark calculation. The results described in the paper should present these data on equal footing. One option would be to show the cost of an MP2 calculation using 40 nodes, though that may lead to a conversation focused on the scaling of the employed MP2 implementation. As an alternative, one could include a comment about the best-case scenario of linear scaling across 40 nodes. In such a case, the MP2 calculation time could be projected to be ~4 hours. That would lead to a different speed-up afforded by GAMA than the 164-fold speed-up reported in the manuscript.***

Response to comment 3 : We thank the reviewer for this insightful suggestion. We have incorporated this suggestion now in the revised manuscript, which can be found on **page 9 (2nd paragraph)** of the revised manuscript, which is highlighted in yellow.

In this work, canonical MP2 is employed for both the supersystem and the GAMA2 fragment calculations to ensure a consistent and unbiased comparison. Under ideal linear scaling scenario, the supersystem MP2 wall time for Alpha-helix-Ala_{18} could be projected to ~4.8 hours on 40 nodes (i.e., 192 hours divided by 40), corresponding to an approximately five-fold speed-up as achieved by GAMA. In practice, however, parallel efficiency in canonical MP2 is limited by its formal $O(N^5)$ scaling, expensive integral transformations, inter-node communication overhead, and load imbalance. Linear-scaling variants of MP2 such as RI-MP2[15–18] and local-MP2[19] significantly reduce computational cost but rely on additional approximations. RI-MP2[15,16] replaces four-center integrals with density-fitted representations using an auxiliary basis set, introducing small but nonzero errors that depend on auxiliary basis quality. Local-MP2[19] further exploits spatial locality by truncating weak orbital-pair correlations, which can reduce accuracy for extended or electronically delocalized systems where correlation effects are inherently nonlocal. To avoid conflating fragmentation errors with approximations from linear-scaling MP2 methods, canonical MP2 is deliberately employed as a common reference. Future work will incorporate RI-MP2 and local-MP2 consistently for both supersystem and fragment

calculations to enable a balanced assessment of computational efficiency and scalability within the GAMA2 framework.

4. ***It seems that MFCC and FBQC may be used in the manuscript without providing their definitions, which should be addressed.***

   Response to comment 4: We thank the reviewer for pointing this out. In the revised manuscript, we have provided the full names and definitions of the acronyms MFCC (Molecular Fractionation with Conjugate Caps) and FBQC (Fragment-Based Quantum Chemistry) at their first occurrence to ensure clarity for the readers.

5. ***Figure 3 shows data for three different systems, but the captions refers to two systems.***

   Response to comment 5: We sincerely thank the reviewer for pointing out this. We have fixed this in the revised manuscript and corrected the caption of **Figure 3**.

Reviewer :3

Review of the manuscript "GAMA: A Robust and Automated Fragment-Based Quantum Chemistry Method for Biomolecular Systems" by Kundu and Saha. The paper presents accuracy and computational benchmarks for GAMA (Grid-Adapted Many-Body Analysis) on biomolecular systems. GAMA performs full-system low-level calculations (HF or DFT) and high-level (MP2) calculations on H-capped fragments, which are automatically prepared based on the selected grid size. The benchmark systems include medium-size polypeptides (including 1YlP, beta-strand Ala18, alpha-helix Ala18, Gly12) with different H-bonding patterns and polarization. The accuracy tests demonstrate that GAMA can reproduce full-system MP2 results with errors up to 13 kcal/mol when HF is used as the full-system low-level method, and 4-5 kcal/mol when DFT functionals are used as the low-level method. The authors indirectly compare GAMA's accuracy with that of other fragmentation methods (MFCC, FMO, SMF, MBE) and claim that GAMA outperforms them. No direct comparisons are shown. Overall, while the development of an accurate and universal fragmentation model is undoubtedly of great demand, the present manuscript does not present sufficient evidence that GAMA will fill this gap. The specific comments are below.

Response: We sincerely thank the reviewer for the detailed evaluation of our manuscript and providing suggestions to improve the clarity of the manuscript. We have addressed all specific comments of the reviewer below.

Addressing specific comments of reviewer 3

1. ***GAMA is limited to mid-system sizes because it is based on full-system calculations with HF or DFT, whereas the discussed fragmentation methods do not have this limitation and have been used to investigate much larger, physically and biologically relevant systems. It would be fairer to compare the performance and accuracy of GAMA with other methods based on the full-system HF method, such as SAPT versions (FSAPT, XSAPT) or ONIOM models.***

   Response to the comment 1: We sincerely thank the reviewer for this thoughtful comment. We acknowledge that, in its current implementation, GAMA is primarily applied to medium-sized systems, as it relies on a full-system HF or DFT reference calculation. The primary objective of the present work is not to claim universal scalability, but to demonstrate that the GAMA protocol—previously developed and validated for noncovalently bound water clusters—can be successfully extended to covalently bonded peptide systems, where accurate total energies are the focus. Here, we report error values from other fragment-based approaches only to provide readers with a reference for assessing GAMA's performance and potential. A system-by-system explicit comparison with other FBQC methods will be addressed in a future, full-length study from our laboratory. Regarding full-system calculations, there are several strategies through which GAMA can be extended to significantly larger systems. Details on these strategies are provided in the "**Future Perspectives**" section on **page 6** of the Supporting Information (SI), where we have highlighted the specific changes for clarity, which we have also provided below.

   There are several strategies, which we are also planning to implement in our future work to reduce or avoid full-system HF calculations in GAMA protocol. For instance, one approach is "forced overlapping fragment generation" through systematic merging of adjacent grids followed by the application of MOBE. Another way is to combine this grid merging protocol with electrostatic charge embedding variant of GAMA[11]. Further strategies under consideration include:

1. **Layered Theoretical Models based on Interaction Proximity:** Within a fixed n-body expansion, the level of theory decreases as the interaction cutoff radius increases. For instance, with a desired 6 Å total interaction cutoff radius, interactions can be treated

hierarchically: CCSD(T) for fragments within 2 Å, MP2 up to 3 Å, B3LYP up to 4 Å, HF up to 5 Å, and semi-empirical PM6 for the remaining fragments up to 6 Å.

2. **Multi-level Treatment of n-body Interactions:** Within a fixed interaction cutoff, the level of theory is scaled inversely with the order of the n-body expansion. For example, at a 5 Å cutoff, 2-body interactions are treated with MP2, while 3-body and 4-body terms are assigned to the more computationally efficient HF and PM6 methods, respectively.

These advanced approaches promise to capture medium- to long-range interactions efficiently, reducing dependence on a single low-level theory. Consequently, GAMA could potentially be applied to extremely large biomolecular systems, including those exceeding 10,000 atoms. Finally, we are exploring integration with machine learning approaches to address persistent challenges in FBQC, such as redundant fragment calculations, accurate treatment of long-range inter-fragment interactions and to develop efficient and user-friendly fragmentation protocols. Together, these strategies highlight the long-term potential of GAMA as a systematically improvable and widely applicable framework for large-scale, accurate quantum chemical calculations on biomolecular systems.

With respect to the suggested comparison to **SAPT**-based methods[20–22] (FSAPT, XSAPT), we note that these approaches are fundamentally designed to compute interaction energies between noncovalently bound subsystems, and have been extensively applied to weakly interacting molecular complexes, molecular clusters. However, SAPT methods do not directly yield total system energies and are not naturally formulated for fully covalently bonded peptide chains, where defining physically meaningful SAPT fragments across covalent bonds is nontrivial. Consequently, SAPT-based approaches are not directly comparable to GAMA in the present context, where total and relative energies of covalently bonded peptides are the quantities of interest. Similarly, ONIOM-based multilayer approaches[23,24] rely on predefined high- and low-level regions, and their accuracy depends sensitively on the choice of layers and the treatment of the interface between them, often involving link atoms and associated boundary artifacts. While ONIOM provides a practical route to reduce computational cost, it does not constitute a systematic many-body reconstruction of the full-system energy, and therefore differs conceptually from the GAMA framework.

Finally, we note that the present contribution is a Letter, and thus subject to strict limitations on scope and length, which preclude the opportunity of extensive side-by-side explicit

benchmarking against alternative FBQC methodologies. The goal of this work is to establish the feasibility, systematic nature, and accuracy trends of GAMA for covalently bonded peptide systems, rather than to claim superior performance over all existing fragmentation approaches. Comprehensive benchmarking against other methods represents an important future direction and will be pursued in dedicated separate future studies.

2. ***It is unclear how GAMA could be applied to any properties rather than total system energies (interaction energies, gradients?). What are the examples of biological problems that GAMA could solve?***

   Response to comment 2: We thank the reviewer for this important point. In the present work, GAMA has been applied and validated only for total energy calculations of peptide systems of varying sizes. The primary goal of this study is to compute the total energies of peptides of different sizes; properties such as interaction energies, energy gradients, or geometry optimizations are not addressed here. The GAMA framework is based on a systematic many-body fragmentation of the total energy and is not inherently limited to energy calculations. In principle, it can be extended to compute energy gradients, perform geometry optimizations, or evaluate interaction energies, provided that the corresponding fragment-level quantities are available. The implementation and validation of these extensions will be pursued systematically in near future works.

   Regarding the detailed biological applications of the GAMA protocol, we have added a **"Future Perspective"** section in the revised Supporting Information, which can be found on **page 6** of revised SI, which has been highlighted in yellow for easy reference, specific part of that Future Perspective section, highlighting the biological applicability of GAMA is provided below.

   In the present work, GAMA has been applied and validated only for medium-sized peptide systems, with a focus on evaluating total energies. While this represents an important proof-of-concept, the framework is inherently flexible and holds significant potential for extension to larger and more complex biomolecular systems. One promising direction is the integration of electrostatically embedded variant, EE-GAMA[11], which we have introduced in our prior work in which the full-system low-level correction can be replaced by point charges. This modification has the potential to allow the treatment of very large systems, such as protein–protein and protein–ligand complexes, while retaining the systematically improvable nature of the fragmentation

protocol. In addition, we plan to explore another variant of GAMA in future in which strongly overlapping fragments will be generated by merging adjacent grid boxes. This approach further reduces reliance on low-level HF-based corrections and expands the applicability of GAMA to much larger biomolecular assemblies. From a biological perspective, GAMA and its variants are particularly suited for applications where accurate total or relative energies are critical. Potential applications include comparing the relative stabilities of peptide or protein conformations, evaluating the energetic effects of localized mutations, assessing relative binding or association energies of biomolecular fragments, and benchmarking correlated wavefunction methods against DFT for biologically relevant subsystems. Furthermore, GAMA-based approaches could be extended to predict protein–ligand and protein–protein binding affinities, providing a valuable tool for drug discovery and rational biomolecular design. The systematic and modular nature of GAMA makes it a flexible platform for future methodological developments, including the calculation of energy gradients, geometry optimizations, and interaction energies, which are beyond the scope of the present work but represent natural extensions. The implementation and thorough validation of these capabilities will be addressed in future studies, highlighting the potential of GAMA as a general, systematically improvable framework for studying increasingly complex biomolecular systems efficiently and accurately.

3. ***The claim of GAMA being "a practical and transformative tool for performing correlated quantum calculations on biomolecular systems that were previously intractable" is clearly an overstatement. As mentioned above, other fragmentation methods have been applied to significantly larger systems than will ever be possible with GAMA. Another question is whether MP2 provides higher accuracy than the new dispersion-corrected functions. Based on benchmarks on non-covalent databases, this is no longer the case, as MP2 overestimates dispersion interactions. Thus, what is the advantage of performing MP2 on top of DFT instead of using a good DFT as the only method?***

   Response to comment 3: We thank the reviewer for this thoughtful comment and for pointing out that the original wording overstated the scope of the present work.

   We agree that several established fragmentation approaches have already been applied to substantially larger biomolecular systems than those considered here. Accordingly,

we have revised the manuscript to replace this specific line that "GAMA2 is a practical and transformative tool for performing correlated quantum calculations on biomolecular systems that were previously intractable" by "GAMA2 is a practical and systematically improvable framework for correlated wavefunction based calculations on medium-sized biomolecular systems" , which can be found at **Page 10** (**2nd paragraph**) of the revised manuscript. In the revised version, we now describe GAMA more conservatively as a practical and systematically improvable framework for performing correlated wavefunction-based calculations on biomolecular systems. This revised wording more accurately reflects the current scope of the method, which is focused on medium-sized peptide systems, while emphasizing its key strengths, namely systematic control of accuracy and compatibility with correlated electronic structure methods.

Regarding the question of MP2 versus modern dispersion-corrected DFT functionals, we agree that for many noncovalent benchmark databases, MP2 can overestimate dispersion interactions and does not universally outperform state-of-the-art DFT methods. However, the systems considered in the present work are covalently bonded peptide systems, rather than weakly bound noncovalent complexes typically used in dispersion benchmarks. In this context, MP2 is not employed primarily as a dispersion model, but as a post–Hartree–Fock treatment of electron correlation within a covalent framework, capturing short- and medium-range correlation effects along the peptide backbone and between nearby residues. Therefore, conclusions drawn from noncovalent benchmark databases do not directly transfer to peptide systems of the type studied here. In addition, many commonly used semi-local and hybrid density functionals are affected by self-interaction errors, which can become particularly problematic in many-body-based fragmented calculations and in systems containing charged, polar, or highly delocalized electronic states[25–27]. These effects may influence the accuracy of total energies in large biomolecular systems, even when dispersion corrections are included. In this regard, MP2 provides a parameter-free, wavefunction-based description of electron correlation that is not affected by self-interaction error and thus serves as a valuable complementary reference. The motivation for performing MP2 on top of DFT in the present work is therefore not to advocate MP2 as a replacement for modern Density Functional Approximations (DFAs), but to enable access to correlated wavefunction energies for medium-sized, covalently connected peptide systems, for which reliable high-level benchmark data are scarce. The GAMA

fragmentation framework makes such MP2 calculations computationally feasible and allows a meaningful assessment of correlated wavefunction treatments alongside modern DFAs within a unified and systematically improvable protocol, without asserting universal superiority of MP2.

4. ***The exact (rather than implicit) benchmarks of GAMA against other available solutions in the field are necessary for any realistic claims of superior accuracy.***
Response to the comment 4: We thank the reviewer for this important and constructive comment. The primary objective of the present work is to demonstrate that the GAMA protocol, which was previously developed and validated for non-covalently bound systems[1,11], can be successfully extended to covalently bonded peptide systems. The focus of this study is therefore on establishing the applicability, robustness, and systematic nature of the GAMA framework for a new and more challenging class of molecular systems, rather than on claiming superior accuracy relative to other existing fragmentation approaches.

In the manuscript, previously reported error values from the literature for other fragmentation methods applied to related peptide systems were cited solely to provide context and to summarize challenges that have already been documented in prior studies. These literature values were not intended to represent an explicit or implicit benchmark against GAMA, nor to suggest superior performance under equivalent computational conditions. We fully agree that any realistic claim of superior accuracy would require explicit and systematic benchmarking against other available fragmentation protocols, carried out under carefully matched computational conditions. Such comprehensive benchmarks would necessitate substantial additional calculations and detailed analyses, which are beyond the scope and length constraints of a Letter-type journal contribution. Accordingly, comprehensive benchmarking of GAMA against other established fragmentation methods is recognized as an important future direction and will be addressed in dedicated follow-up work. We have reframed the two paragraphs in the revised manuscript where other FBQC methods are mentioned, to clarify that these literature benchmarks are discussed solely to provide context and highlight known challenges in the field, rather than to make a direct comparison or claim superiority of GAMA2, which we have provided below.

One paragraph which can be found on **page 3** of the revised manuscript which is highlighted in yellow and which is also provided below.

GAMA2 adopts an overlapping, grid-defined fragmentation strategy combined with a multilayer framework to address challenges associated with covalently connected biomolecular systems. In contrast to cap-based approaches such as Molecular Fractionation with Conjugate Caps[7,28,29] (MFCC), the method avoids the introduction of artificial caps and the associated boundary effects. Relative to standard nonoverlapping many-body expansion (MBE)–based schemes, the use of overlapping fragments within GAMA2 enables recovery of important delocalization, polarization, and correlation effects without requiring high-order expansions or very large embedding domains. While Fragment-Molecular Orbital (FMO) methods[30,31] efficiently account for polarization, GAMA2's grid-based construction generates overlapping fragments, enabling a more systematic treatment of electronic delocalization. Overall, GAMA2 is intended as an alternative fragmentation framework that emphasizes automation, systematic improvability, and compatibility with correlated wavefunction methods, rather than as a direct comparison or direct replacement for other existing FBQC approaches.

Another paragraph which can be found on **Page 5** of the revised manuscript which is also highlighted in yellow and which is also provided below,

Existing FBQC methods[7,8,10,12,13] have laid the strong foundation of this field in the last three decades providing an opportunity to make these methods more powerful. Towards that direction, we are focusing on existing challenges. Published benchmarks in the literature provide useful context regarding the challenges faced by FBQC methods for covalently bonded peptide systems. For example, Vornweg and co-workers[7] applied conventional MFCC and MFCC-MBE(2) schemes to three Ala_{10} peptide isomers (Alpha-helix, Beta-strand, and 3_{10}-helix) and reported deviations from supersystem MP2 energies: MFCC errors of ~ **60–80 kcal/mol** (~ **6–8 kcal/mol per peptide**) and MFCC-MBE(2) errors of ~ **6–8 kcal/mol** (~ **0.6–0.8 kcal/mol per peptide**) for the Alpha-helix and 3_{10}-helix isomers. Similar trends have been observed for Fragment Molecular Orbital (FMO) methods[14], which improve upon MFCC for polar or charged peptides but still show deviations of ~ **10–16 kcal/mol** (~ **0.5–2 kcal/mol per peptide**) for FMO2 and ~ **5–8 kcal/mol** (~ **0.2–0.8 kcal/mol per peptide**) for FMO3 in Alpha-

helical polyalanine chains of 10–20 residues. SMF and MBE-with-capping approaches also show deviations of ~ **8–20 kcal/mol** (~ **0.8–2 kcal/mol per peptide**) for compact or Alpha-helical structures, reflecting incomplete recovery of higher-order polarization, charge-transfer, and cooperative hydrogen-bonding effects. These studies additionally highlight the considerable computational cost of several FBQC approaches[8–10]—particularly higher-order MBE, FMO3, and large-cutoff Systematic Molecular Fragmentation (SMF) approaches—which can require hundreds to thousands of CPU hours for 15–20 residue peptides. Importantly, because these published benchmarks employ varying fragmentation protocols and reference levels of theory, they do not allow for a direct side-by-side comparison with GAMA. Consequently, we do not claim that GAMA offers superior accuracy relative to these established benchmarks. Instead, these literature results serve to contextualize the broader challenges within the field and provide a frame of reference for our method's performance. Within this context, GAMA2 achieves total energy deviations of **0.01–4 kcal/mol** for flexible peptides (HF low-level) and **2–5 kcal/mol** for highly cooperative Alpha-helical-Ala_{18} and 3_{10}-helical-Ala_{18} systems (DFT low-level). Beyond accuracy, GAMA2 significantly optimizes efficiency: a full MP2 calculation for Alpha-helix-Ala_{18} peptide system requires ~**1,178 CPU hours**, whereas GAMA2 completes the task in just **~77 CPU hours** (**Figure S2**, **Table S7**). These results position GAMA2 as a systematically improvable fragmentation scheme that complements existing Fragment-Based Quantum Chemistry (FBQC) methods for covalently connected biomolecules.

References

(1) Kundu Sujan, S. A. Grid-Adapted-Manybody-Analysis (GAMA): A Fragment-Based Quantum Chemistry Exploration on Medium-Sized Water Clusters and the Critical Role of Counterpoise Correction. *Chem Rxiv* **2025**, No. 10.26434/chemrxiv-2025-z5115 D O I: 10.26434/chemrxiv-2025-z5115.

(2) Nguyen, A. L. P.; Izgorodina, E. I. Behavior of Counterpoise Correction in Many-Body Molecular Clusters of Organic Compounds: Hartree–Fock Interaction Energy Perspective. *J. Comput. Chem.* **2022**, *43* (8), 568–576. https://doi.org/10.1002/jcc.26814.

(3) Gray, M.; Bowling, P. E.; Herbert, J. M. Systematic Evaluation of Counterpoise Correction in Density Functional Theory. *J. Chem. Theory Comput.* **2022**, *18* (11), 6742–6756. https://doi.org/10.1021/acs.jctc.2c00883.

(4) Boys S.F, B. F. The Calculation of Small Molecular Interactions by the Differences of Separate Total Energies. Some Procedures with Reduced Errors. *Mol. Phys.* **1970**, 553–566.

(5) Valiron Pierre, M. I. Elsevier Chemical Physics Letters Volume 275, Issues 1–2, 22 August 1997, Pages 46-55 Chemical Physics Letters Hierarchy of Counterpoise Corrections for N-Body Clusters: Generalization of the Boys-Bernardi Scheme. *Chem. Phys. Lett.* **1997**, *275* (1–2), 46–55.

(6) Liu, J.; Herbert, J. M. Pair-Pair Approximation to the Generalized Many-Body Expansion: An Alternative to the Four-Body Expansion for Ab Initio Prediction of Protein Energetics via Molecular Fragmentation. *J. Chem. Theory Comput.* **2016**, *12* (2), 572–584. https://doi.org/10.1021/acs.jctc.5b00955.

(7) Vornweg, J. R.; Wolter, M.; Jacob, C. R. A Simple and Consistent Quantum-Chemical Fragmentation Scheme for Proteins That Includes Two-Body Contributions. *J. Comput. Chem.* **2023**, *44* (18), 1634–1644. https://doi.org/10.1002/jcc.27114.

(8) Raghavachari, K.; Saha, A. Accurate Composite and Fragment-Based Quantum Chemical Models for Large Molecules. *Chem. Rev.* **2015**, *115* (12), 5643–5677. https://doi.org/10.1021/cr500606e.

(9) Saha, A.; Raghavachari, K. Analysis of Different Fragmentation Strategies on a Variety of Large Peptides: Implementation of a Low Level of Theory in Fragment-Based Methods Can Be a Crucial Factor. *J. Chem. Theory Comput.* **2015**, *11* (5), 2012–2023. https://doi.org/10.1021/ct501045s.

(10) Herbert, J. M. Fantasy versus Reality in Fragment-Based Quantum Chemistry. *J. Chem. Phys.* **2019**, *151* (17), 1–38. https://doi.org/10.1063/1.5126216.

(11) Kundu, S.; Saha, A. Electrostatically Embedded Grid-Adapted Many-Body Analysis (EE-GAMA): A Charge Embedded Fragment-Based Quantum Chemistry Method for Accurate Modelling of Neutral and Charged Molecular Clusters. *Comput. Theor. Chem.* **2025**, *1254* (August), 115534. https://doi.org/10.1016/j.comptc.2025.115534.

(12) Collins, M. A.; Bettens, R. P. A. Energy-Based Molecular Fragmentation Methods. *Chem. Rev.* **2015**, *115* (12), 5607–5642. https://doi.org/10.1021/cr500455b.

(13) Gordon, M. S.; Fedorov, D. G.; Pruitt, S. R.; Slipchenko, L. V. Fragmentation Methods: A Route to Accurate Calculations on Large Systems. *Chem. Rev.* **2012**, *112* (1), 632–672. https://doi.org/10.1021/cr200093j.

(14) Vuong, V. Q.; Nishimoto, Y.; Fedorov, D. G.; Sumpter, B. G.; Niehaus, T. A.; Irle, S.; Villeurbanne, F.-. The Fragment Molecular Orbital Method Based on Long-Range

Corrected Density-Functional Tight-Binding. **2019**. https://doi.org/10.1021/acs.jctc.9b00108.

(15) Snowdon, C.; Barca, G. M. J. An Efficient RI-MP2 Algorithm for Distributed Many-GPU Architectures. **2024**. https://doi.org/10.1021/acs.jctc.4c00814.

(16) Weigend, F.; Häser, M.; , Holger Patzelt, R. A. RI-MP2: Optimized Auxiliary Basis Sets and Demonstration of Efficiency. *Chem. Phys. Lett.* **1998**, *294* (1–3), 143–152.

(17) You, A.; Be, M. A. Y.; In, I. Laplace Transform Techniques in Mo / Ller – Plesset Perturbation Theory. **2008**, *489* (June 1998).

(18) Del Ben M, Hutter J, V. J. Probing the Structural and Dynamical Properties of Liquid Water with Models Including Non-Local Electron Correlation. *J. Chem. Phys.* **2015**, *143* (5).

(19) Maschio, L.; Usvyat, D.; Manby, F. R.; Casassa, S.; Pisani, C.; Schütz, M. Fast Local-MP2 Method with Density-Fitting for Crystals . I . Theory and Algorithms. **2007**, 1–9. https://doi.org/10.1103/PhysRevB.76.075101.

(20) Lao, K. U.; Herbert, J. M. Accurate and E Ffi Cient Quantum Chemistry Calculations for Noncovalent Interactions in Many-Body Systems : The XSAPT Family. **2014**.

(21) Hapka, M.; Przybytek, M.; Pernal, K. Symmetry-Adapted Perturbation Theory Based on Multicon Fi Gurational Wave Function Description of Monomers. **2021**. https://doi.org/10.1021/acs.jctc.1c00344.

(22) Herbert, J. M.; Gray, M.; Carter-fenk, K. Extended Symmetry-Adapted Perturbation Theory (XSAPT): A Cubic-Scaling Platform for Computing Accurate Intermolecular Interaction Energies and Ab Initio Energy Decomposition Analysis. **2026**, No. September 2025, 137–222. https://doi.org/10.1007/430.

(23) Chung, L. W.; Sameera, W. M. C.; Ramozzi, R.; Page, A. J.; Hatanaka, M.; Petrova, G. P.; Harris, T. V.; Li, X.; Ke, Z.; Liu, F.; Li, H. B.; Ding, L.; Morokuma, K. The ONIOM Method and Its Applications. *Chem. Rev.* **2015**, *115* (12), 5678–5796. https://doi.org/10.1021/cr5004419.

(24) Svensson, M.; Humbel, S.; Froese, R. D. J.; Matsubara, T.; Sieber, S.; Morokuma, K. ONIOM: A Multilayered Integrated MO + MM Method for Geometry Optimizations and Single Point Energy Predictions. A Test for Diels-Alder Reactions and Pt(P(t-Bu)3)2 + H2 Oxidative Addition. *J. Phys. Chem.* **1996**, *100* (50), 19357–19363. https://doi.org/10.1021/jp962071j.

(25) Article, E.; Herbert, J. M. Chemical Science Many-Body Expansion †. **2024**, 19893–19906. https://doi.org/10.1039/d4sc05955g.

(26) Broderick, D. R.; Herbert, J. M. Untangling Sources of Error in the Density-Functional Many-Body Expansion. **2025**. https://doi.org/10.1021/acs.jpclett.4c03619.

(27) Bao, J. L.; Gagliardi, L.; Truhlar, D. G. Self-Interaction Error in Density Functional Theory : An Appraisal. **2018**, 6–11. https://doi.org/10.1021/acs.jpclett.8b00242.

(28) Mei, Y.; Ji, C.; Zhang, J. Z. H. A New Quantum Method for Electrostatic Solvation Energy of Protein. *J. Chem. Phys.* **2006**, *125* (9). https://doi.org/10.1063/1.2345201.

(29) Vornweg, J. R.; Jacob, C. R. Protein − Ligand Interaction Energies from Quantum-Chemical Fragmentation Methods: Upgrading the MFCC-Scheme with Many- Body Contributions. **2024**. https://doi.org/10.1021/acs.jpcb.4c05645.

(30) Fedorov, D. G.; Ishida, T.; Kitaura, K. Multilayer Formulation of the Fragment Molecular Orbital Method (FMO). *J. Phys. Chem. A* **2005**, *109* (11), 2638–2646. https://doi.org/10.1021/jp047186z.

(31) Fedorov, D. G.; Ishida, T.; Uebayasi, M.; Kitaura, K. The Fragment Molecular Orbital Method for Geometry Optimizations of Polypeptides and Proteins. *J. Phys. Chem. A* **2007**, *111* (14), 2722–2732. https://doi.org/10.1021/jp0671042.

jz-2025-03778a.R2

Name: Peer Review Information for "GAMA: A Robust and Automated Fragment-Based Quantum Chemistry Method for Biomolecular Systems"

Second Round of Reviewer Comments

Reviewer: 2

Comments to the Author

I thank the authors for their careful consideration of my comments. The review responses and manuscript edits address my prior concerns.

Reviewer: 1

Comments to the Author

The authors have addressed comments made in the previous round of reviews. These revisions have added context and clarified some of the technical aspects of the method. I would recommend this letter for publication in JPC Letters after addressing the minor points below.

- The discussion starting on Pg. 2 l. 22 covers two topics: an overview of the GAMA algorithm and how the GAMA algorithm is applied to biomolecular systems. This discussion may be clearer if the if the general algorithm (previous work) and the bio-specific application (this letter) were separated into two separate paragraphs.

- On Pg. 2 l. 50 the using "interaction distance cutoff" or "radial cutoff" may be less ambiguous than "interaction cutoff" since other metrics, such as interaction energy, may be used.

- SMF is used on Pg. 5 before it is defined on Pg. 6

- On Pg. 9, the authors rightly explain that GAMA (and other fragment methods) are often more efficient because the polynomial scaling fragment calculations are much smaller. This point may be made more explicit stating the worst-case scaling for GAMA is N_F O(n5) where N_F is the number of fragments and n is the size of your largest fragment. Also note that O in big-O notation on Pg. 9 l. 48 should be italicized.

Author's Response to Peer Review Comments:

Response to the reviewer (Second round of revision)

We sincerely thank all the reviewers for their careful evaluation of our manuscript and for their insightful and constructive comments, which have significantly helped to improve the quality of our work. We have addressed all the comments from both of the reviewers and incorporated the necessary revisions into the manuscript.

Reviewer: 1

Recommendation: This paper is publishable subject to minor revisions noted. Further review is not needed.

Comments: The authors have addressed comments made in the previous round of reviews. These revisions have added context and clarified some of the technical aspects of the method. I would recommend this letter for publication in JPC Letters after addressing the minor points below.

- The discussion starting on Pg. 2 l. 22 covers two topics: an overview of the GAMA algorithm and how the GAMA algorithm is applied to biomolecular systems. This discussion may be clearer if the general algorithm (previous work) and the bio-specific application (this letter) were separated into two separate paragraphs.

- On Pg. 2 l. 50 the using "interaction distance cutoff" or "radial cutoff" may be less ambiguous than "interaction cutoff" since other metrics, such as interaction energy, may be used.

- SMF is used on Pg. 5 before it is defined on Pg. 6

- On Pg. 9, the authors rightly explain that GAMA (and other fragment methods) are often more efficient because the polynomial scaling fragment calculations are much smaller. This point may be made more explicit stating the worst-case scaling for GAMA is N_F O(n5) where N_F is the number of fragments and n is the size of your largest fragment. Also note that O in big-O notation on Pg. 9 l. 48 should be italicized.

Response: We sincerely thank the reviewer for thorough evaluation manuscript and providing constructing comments which significantly enhance the quality of the present manuscript. We have addressed all the specific comments of the reviewer below.

1. ***The discussion starting on Pg. 2 l. 22 covers two topics: an overview of the GAMA algorithm and how the GAMA algorithm is applied to biomolecular systems. This***

discussion may be clearer if the general algorithm (previous work) and the bio-specific application (this letter) were separated into two separate paragraphs.

Response to comment 1: We thank the reviewer for this helpful suggestion. The discussion has been reorganized into two separate paragraphs to clearly distinguish (i) the general GAMA framework and its conceptual extension introduced in this work and (ii) the specific implementation of the method for covalently bonded peptide systems. Which can be found at page 2 of the revised manuscript, which is also highlighted in yellow. We have also provided two separate paragraphs below for further clarification.

Paragraph 1: In our previous work, we introduced the Grid-Adapted Many-Body Analysis (GAMA) framework[7], along with its charge-embedded extension, EE-GAMA[8], to enable accurate and efficient fragment-based quantum calculations on molecular systems, particularly those dominated by noncovalent interactions. The original GAMA[7] framework was systematically validated for noncovalent water clusters. In this approach, the entire system is first enclosed within a three-dimensional spatial box, which is subsequently partitioned into smaller grid cells. Each water molecule is treated as an indivisible unit to preserve its internal structure during fragmentation. Based on this grid partitioning, primary fragments are constructed from molecules residing within a given grid, while overlapping fragments are generated by including molecules at the interfaces of neighbouring grids. Treating the primary and overlapping fragments as monomeric units, their energies are evaluated within the Many Overlapping Body Expansion[9] (MOBE) framework, truncated at the two-body term. To capture residual long-range many-body effects, a two-layer correction scheme—analogous to the MIM[10] or ONIOM[11]-type approach—is incorporated, enabling an accurate reconstruction of the total system energy from the fragment contributions. The EE-GAMA[8] framework extends this methodology by introducing electrostatic embedding, wherein each fragment is computed in the presence of background point charges that represent the electrostatic influence of the surrounding molecular environment. This inclusion allows the fragment calculations to capture polarization and other environment-dependent effects that are otherwise neglected in non-embedded GAMA. EE-GAMA has been successfully applied to both neutral and protonated water clusters (hydronium cluster systems), demonstrating a significant enhancement in accuracy compared to the original, non-embedded GAMA scheme. By systematically combining fragment-based expansion with electrostatic embedding, EE-

GAMA provides a robust and scalable approach for high-precision quantum chemical calculations of large, noncovalently bound molecular assemblies.

Paragraph 2: In this present work, we generalize this approach to covalently bonded peptide systems. This extension preserves the simplicity and physical transparency of the original method while enabling grid-based fragmentation at correlated levels of theory. The major goal of this work is to develop a fully automated and systematically improvable fragment-based quantum chemical framework applicable to covalently bonded biomolecular systems, where fragmentation across covalent bonds poses a fundamental challenge. Building on our earlier Grid-Adapted Many-Body Analysis (GAMA) framework developed for noncovalently bound water clusters, we extend the GAMA protocol to covalently bonded peptide systems. The approach combines a grid-based fragmentation scheme, a many-body expansion truncated at the two-body level, and a low-level correction. The major impact of this work is the demonstration that GAMA2 (GAMA with two different layers) provides a controllable and efficient FBQC approach whose performance can be systematically tuned through the choice of low-level theory, grid box size, and interaction distance cutoff, enabling accurate MP2-level energies at substantially reduced computational cost and significantly advancing fragment-based treatments of large biomolecular systems. The practical implementation of this GAMA framework for covalently bonded peptide systems is described as follows. To construct peptide fragments, the full structure is enclosed in a simulation box defined by its cartesian extents and partitioned into smaller cubic grid cells. In parallel, the peptide backbone is segmented by cleaving the relatively less polar C–C single bond (across the peptide backbone) between the C-alpha of residue *i* and the carbonyl carbon of residue *i+1*, with broken valencies saturated using standard hydrogen link atoms[12–14]. This avoids cutting through the polar peptide bond and eliminates the need for the more complex capping strategies used in MFCC[15,16]-type schemes. Here we also avoid cutting the C-C bond at the side chain of the peptide. Herbert and co-workers (Ref. 14) highlighted the advantages of cutting the C–C single bond between alpha carbon and carbonyl carbon along the peptide backbone while retaining the polar peptide and N–C-alpha bonds. Each "group of atoms" (unbreakable segment) is mapped onto the grid; groups occupying a given grid cell define primary fragments. Because a group may span adjacent cells, overlapping fragments arise naturally—an essential feature of GAMA. These primary and overlapping fragments

constitute the monomers for the Many-Overlapping Body Expansion[9] (MOBE), truncated here at the two-body level to balance accuracy and efficiency. Long-range electrostatics are recovered via a multilayer ONIOM[11,17]-like correction: fragment interactions are computed at the MP2/6-311G(d,p) level within MOBE, while a full-system HF/6-311G(d,p) calculation supplies complementary long-range contributions. This multilayer strategy, established in our earlier work[7,8], provides a systematically improvable treatment of both short- and long-range correlation effects. We refer to the present two-level formulation as GAMA2. Full methodological and mathematical details are provided in our earlier publications[7,8].

2. ***On Pg. 2 l. 50 the using "interaction distance cutoff" or "radial cutoff" may be less ambiguous than "interaction cutoff" since other metrics, such as interaction energy, may be used.***

   Response to comment 2: We sincerely thank the reviewer for this comment,which provide more clarification about our method. We have now fixed this in the revised manuscript

3. ***SMF is used on Pg. 5 before it is defined on Pg. 6***

   Response to comment 3: We sincerely thank the reviewer for this comment. Now in the revised manuscript we have fixed this.

4. ***On Pg. 9, the authors rightly explain that GAMA (and other fragment methods) are often more efficient because the polynomial scaling fragment calculations are much smaller. This point may be made more explicit stating the worst-case scaling for GAMA is N_F O(n5) where N_F is the number of fragments and n is the size of your largest fragment. Also note that O in big-O notation on Pg. 9 l. 48 should be italicized.***

   Response to comment 4: We sincerely thank the reviewer for this helpful suggestion. We have revised the manuscript (Pg. 9) to explicitly state that the worst-case computational scaling of GAMA can be expressed as $N_F * O(n^5)$, where $N_F$denotes the number of fragments and n represents the size of the largest fragment. This clarification makes the computational advantage of the method more transparent. In addition, we have corrected the formatting of the big-O notation on Pg. 9, line 48, and italicized the O to ensure consistency with standard mathematical conventions. We have provided the specific paragraph below , which is also highlighted in yellow

It is an important point to note that the formal computational scaling of GAMA calculations using MP2 can be expressed as $N_F * \mathcal{O}(n^5)$, where N_F is the number of fragments and n denotes the size of the largest fragment.

Reviewer: 2

Recommendation: This paper represents a significant new contribution and should be published as is.

Comments: I thank the authors for their careful consideration of my comments. The review responses and manuscript edits address my prior concerns.

Response: We sincerely thank the reviewer for thorough evaluation of our manuscript and recommend for publication of our manuscript in Journal of Physical Chemistry Letters (JPCL)
